# Cytoplasmic accumulation and plasma membrane association of anillin and Ect2 promote confined migration and invasion

**DOI:** 10.21203/rs.3.rs-3640969/v1

**Published:** 2024-01-10

**Authors:** Avery T. Tran, Emily O. Wisniewski, Panagiotis Mistriotis, Konstantin Stoletov, Maria Parlani, Alice Amitrano, Brent Ifemembi, Se Jong Lee, Kaustav Bera, Yuqi Zhang, Soontorn Tuntithavornwat, Alexandros Afthinos, Alexander Kiepas, John J. Jamieson, Yi Zuo, Daniel Habib, Pei-Hsun Wu, Stuart S. Martin, Sharon Gerecht, Luo Gu, John D. Lewis, Petr Kalab, Peter Friedl, Konstantinos Konstantopoulos

**Affiliations:** 1 Department of Chemical and Biomolecular Engineering, The Johns Hopkins University, Baltimore MD, 21218, USA; 2 Johns Hopkins Institute for NanoBioTechnology, The Johns Hopkins University, Baltimore MD, 21218, USA; 3 Department of Chemical Engineering, Auburn University, Auburn, AL, 36849, USA; 4 Department of Oncology, University of Alberta, Edmonton, AB T6G 2E1, Canada; 5 Department of Medical Biosciences, Radboud University Medical Center, Nijmegen, Netherlands; 6 Department of Materials Science and Engineering, The Johns Hopkins University, Baltimore MD, 21218, USA; 7 Marlene and Stewart Greenebaum National Cancer Institute Comprehensive Cancer Center, University of Maryland School of Medicine, Baltimore, MD, 21201, USA; 8 Department of Pharmacology, University of Maryland School of Medicine, Baltimore, MD, 21201, USA; 9 Department of Biomedical Engineering, Duke University, Durham, NC 27708, USA; 10 Department of Genitourinary Medicine, UT MD Anderson Cancer Center, Houston TX, 77030 USA; 11 Department of Biomedical Engineering, The Johns Hopkins University, Baltimore MD, 21218, USA; 12 Department of Oncology, The Johns Hopkins University, Baltimore MD, 21205, USA

## Abstract

Cells migrating in confinement experience mechanical challenges whose consequences on cell migration machinery remain only partially understood. Here, we demonstrate that a pool of the cytokinesis regulatory protein anillin is retained during interphase in the cytoplasm of different cell types. Confinement induces recruitment of cytoplasmic anillin to plasma membrane at the poles of migrating cells, which is further enhanced upon nuclear envelope (NE) rupture(s). Rupture events also enable the cytoplasmic egress of predominantly nuclear RhoGEF Ect2. Anillin and Ect2 redistributions scale with microenvironmental stiffness and confinement, and are observed in confined cells *in vitro* and in invading tumor cells *in vivo*. Anillin, which binds actomyosin at the cell poles, and Ect2, which activates RhoA, cooperate additively to promote myosin II contractility, and promote efficient invasion and extravasation. Overall, our work provides a mechanistic understanding of how cytokinesis regulators mediate RhoA/ROCK/myosin II-dependent mechanoadaptation during confined migration and invasive cancer progression.

Cancer cell migration through confined spaces is a critical step in the metastatic process^1^. To colonize distant organs, cells escaping from the primary tumor must successfully navigate pores in the extracellular matrix (ECM) as well as 3-dimensional (3D) longitudinal tissue tracks, which exist naturally between various anatomical structures or can be created de-novo by matrix remodeling of dense ECM by stromal cells or tumor cells themselves^1^. Such paths impose varying degrees of confinement, as cells must travel through confining pores ranging from 1 to 20 μm in diameter, or fiber- and channel-like tracks varying from less than 3 up to 30 μm in width^2^. During extravasation, tumor cells must also transmigrate through narrow gaps between adjacent endothelial cells ranging from 2–5 μm in diameter^3^. Physical cues, such as confinement, initiate intracellular signaling cascades that enable cells to adapt their migration mechanisms and modes to their microenvironment^4^.

Rho GTPases, which are overexpressed in human tumors^5^, play a pivotal role in regulating confined cell migration^1, 6^. While Rac1 promotes actin polymerization and the formation of lamellipodia protrusions typically associated with a mesenchymal migration mode that has commonly been observed on 2D substrates, Rho GTPase signaling also regulates other distinct migration modes in 3D microenvironments^7, 8^. Bleb-based migration, which is characterized by spherical membrane protrusions produced by contractions of the actomyosin cortex^9^, is one such migration phenotype prompted by RhoA/ROCK activity^10^ when cell-matrix adhesion is low. Bleb-based migration is often induced by extracellular protease inhibition^10^ and can allow cells to squeeze through gaps in the extracellular matrix^9^. Different amoeboid modes of migration are characterized by stable bleb formation^11^. In addition to bleb-based migration, elevated RhoA-dependent contractility promotes lobopodial-based migration by facilitating nuclear pulling^12^. Furthermore, distinct spatial regulation of Rho GTPases can tune the migration mode in confinement^13^. In conjunction with Rac1-driven lamellipodia or filopodia formation at the cell leading edge, RhoA/ROCK orchestrates translocation of the cell rear in matrix-directed cell migration^14^.

In addition to regulating the migration phenotype, confinement promotes nuclear bleb formation and rupture^15–17^. NE rupture enables the rapid exchange of material between the nuclear and cytoplasmic compartments that can persist for hours^15–17^. However, the identity and function of nuclear proteins escaping to the cytoplasm during NE rupture in confined cell migration remain unclear. Among the proteins that are predominantly localized in the interphase nuclei due to energy-dependent RanGTPase- and importin-mediated nuclear import are mitotic regulators, including the cytokinesis regulators anillin and Ect2, both of which are directly involved in the localized RhoA activation at the plasma membrane^18–21^.

Besides confined cell migration, Rho GTPase regulation is essential during cell division, specifically during cytokinesis, when RhoA acts as a critical regulator of actomyosin-based contractile ring formation and ingression^22^. During cytokinesis, RhoA is directly activated by the RhoGEF Ect2^21^, which gets anchored to the equatorial cortex via its interaction with anillin^23^, a scaffolding protein that binds to plasma membrane and links RhoA, actin, and myosin^24^. Several phenotypic parallels exist between RhoA/myosin II-dependent contractility activation during cytokinesis and confined cell migration, including polarized distributions of myosin II and actin, colocalization of RhoA, actin, and myosin II^13, 15, 24^, and cytoplasmic bleb formation^9, 13^. In addition to their primary role in cell cytokinesis, recent evidence has implicated anillin and Ect2 in tumorigenic and metastatic processes^25, 26^ via mechanisms that are yet to be elucidated. We herein demonstrate that confinement promotes recruitment of the cytoplasmic anillin to the plasma membrane at the front and rear poles of migrating cells, resulting in the formation of anillin-rich zones at cell edges. NE rupture, which frequently occurs during cell entry and migration in confining spaces, further enriches anillin at the cell poles, and also induces Ect2 release into the cytoplasm and its uniform accumulation at the plasma membrane. These data indicate that anillin functions as a critical scaffolding factor mediating the polarized Ect2 and RhoA-dependent actomyosin contractility in migrating cancer cells in confinement, thereby facilitating their invasion and extravasation.

## RESULTS

### Confinement induces switch from protrusive to bleb-based migration by spatially regulating RhoA activity

**Figure 1.**
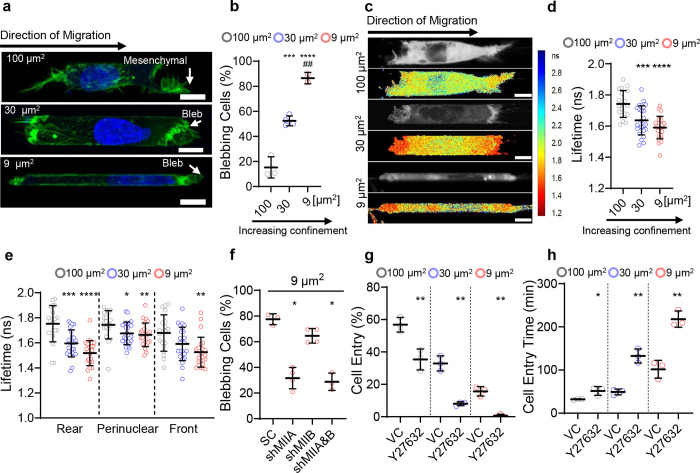
Confinement induces RhoA activation to facilitate cell entry and bleb-based migration. **(a)** Percentage of HT-1080 cells migrating with a bleb-based migration phenotype in 100 μm^2^, 30 μm^2^, and 9 μm^2^ channels (n≥20 cells per experiment from ≥2 experiments). **(b)** Representative images of HT-1080 cell migration phenotype in 100 μm^2^, 30 μm^2^, and 9 μm^2^ channels, as assessed from cells fixed and stained with AF488 phalloidin and Hoechst 33342 (Scale bars: 10 μm). **(c)** Increasing confinement elevates RhoA activity and polarization as measured by FLIM-FRET of RhoA2G biosensor. Representative cells in 100 μm^2^, 30 μm^2^, and 9 μm^2^ microchannels. Gray scale image is donor intensity and heat map illustrates subcellular distribution of activated RhoA. Scale bars: 10μm. **(d)** The donor fluorescence lifetime of RhoA activity biosensor RhoA2G inside 100 μm^2^, 30 μm^2^, and 9 μm^2^ channels, as measured by FLIM-FRET (n≥20 cells from 3 experiments). **(e)** Spatial distribution of RhoA activity in cells migrating inside 100 μm^2^, 30 μm^2^, and 9 μm^2^ microchannels as measured by FLIM-FRET (n≥20 cells from 3 experiments). **(f)** Percentage of scramble control (SC), MIIA, MIIB, or dual MIIA and MIIB knockdown HT-1080 cells migrating with a blebbing phenotype in 9 μm^2^ channels (n≥15 cells per experiment from ≥3 experiments). **(g,h)** Percentage of cells that enter (g) and time required for cell entry (h) into 100 μm^2^, 30 μm^2^, and 9 μm^2^ channels and in the presence of Y27632 (10 μM) or vehicle control (VC). Data represent averages per experiment (n≥20 cells per experiment from 3 experiments). Values represent the mean±S.D. *p<0.05, **p<0.01, ***p<0.001, ****p<0.0001 relative to 100 μm^2^ or VC or SC; ##p<0.01 relative to 30 μm^2^ assessed by one-way ANOVA followed by Tukey’s multiple comparisons test (b,d, e (rear)), Kruskal-Wallis followed by Dunn’s multiple comparisons test (e (perinuclear, front), f), unpaired t-test (g (100 μm^2^), h) with Welch’s correction (g (30 and 9 μm^2^)).

To delineate the effect of increasing confinement on cell migration mode and efficiency, we induced HT-1080 fibrosarcoma cells to migrate through moderately-confining (*A*=100 μm^2^, *W(idth)*, *H(eight)*=10 μm), confining (*A*=30 μm^2^, *W*=10 μm, *H*=3 μm), or tightly confining (*A*=9 μm^2^, *W*, *H*=3 μm) collagen-I-coated microchannels. The majority (~85%) of HT-1080 cells in moderately-confining channels exhibited finger-like protrusions consistent with their characteristic mesenchymal phenotype (Fig. 1a,b). As the degree of confinement increased, cells switched from a primarily mesenchymal to bleb-based migration mode (Fig. 1a,b). Blebbing cells displayed a pill-like morphology with membrane blebs, which were identified as sphere-like bulges (Fig. 1a). Confinement-induced cell blebbing was also observed for MDA-MB-231 breast cancer cells and human osteosarcoma (HOS) cells (Suppl. Fig. 1a,b).

Because bleb formation requires the activation of RhoA/myosin-II-dependent contractility^10, 13^, we used confocal fluorescence-lifetime imaging microscopy (FLIM) coupled with a Förster Resonance Energy Transfer (FRET)-based RhoA2G activity biosensor^15, 27^ to quantify RhoA activity. In line with an increase in the blebbing phenotype, cells migrating in confining or tightly confining channels displayed increased RhoA activity, as evidenced by decreased donor fluorescence lifetimes compared to cells in moderately-confining channels (Fig. 1c,d). RhoA activity was polarized with maximal levels detected in areas of membrane blebs at the poles of confined and tightly-confined cells (Fig. 1e). In contrast, cells in moderately-confining channels displayed relatively uniform basal levels of RhoA activity (Fig. 1e). Knockdown of *myosin IIA* (MIIA or MYH9)^15^ converted cells to a predominantly mesenchymal phenotype in tightly confining channels (Fig. 1f) without affecting the migration mode in moderately-confining channels (Suppl. Fig. 1c)^13^. Depletion of *myosin IIB* (MIIB or MYH10)^15^ had no effect on migration phenotype in tight confinement (Fig. 1f). Inhibition of the RhoA/ROCK pathway with Y27632 (10 μM) markedly suppressed cell entry into confining and tightly confining channels, but to a lower extent in moderately-confining ones (Fig. 1g,h). These results reveal that RhoA/ROCK/MIIA-dependent contractility, which converts cells to a bleb-based migration phenotype, becomes elevated in confinement and facilitates cell entry into confined spaces.

In addition to plasma membrane blebbing, confinement, by exerting mechanical stress on the nucleus, promoted nuclear envelope blebbing, which frequently resulted in NE rupture (Suppl. Fig. 1d) and the exchange of contents between the nucleus and the cytoplasm^15–17^. In line with their elongated morphology on 2D, HT-1080 cells initiated entry into PDMS-based confined channels while displaying a mesenchymal migration mode, and the majority transitioned upon channel entry to a bleb-based migration phenotype (Fig. 1b and Suppl. Fig. 1e). The transition of HT-1080 cells from mesenchymal to cytoplasmic blebbing phenotype in confining (30 μm^2^) channels frequently coincided with or followed NE rupture events (Suppl. Fig. 1f). This coincidence of confinement-induced NE rupture and cell blebbing suggested that nuclear constituents escaping to the cytoplasm following nuclear rupture could promote RhoA/myosin II activation, thereby facilitating cell entry into confining spaces. We noted that the polarized patterns of RhoA activity (Fig. 1c,e) along with the coordinated action of RhoA and myosin II observed in confinement are reminiscent of cytokinesis^24^, prompting us to examine the potential role of cytokinesis regulators in confined migration.

### Confinement promotes the accumulation of anillin to the cell poles, which is further enriched by NE rupture

**Figure 2.**
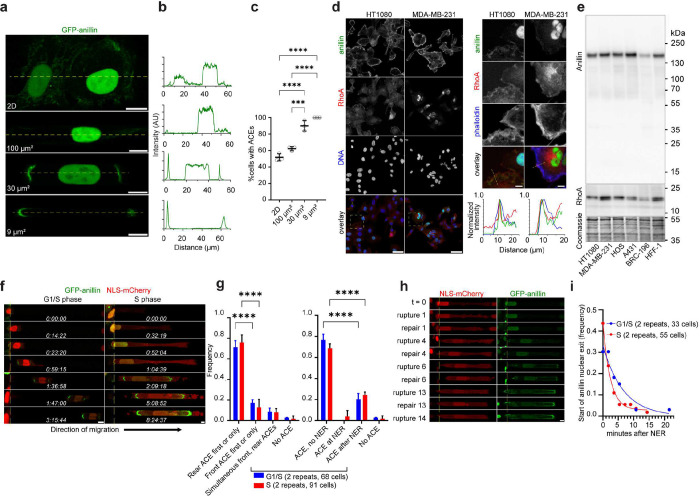
ACEs (Anillin at Cell Edges) exist in cells on 2D and are further enriched in confinement. **(a-c)** Representative live-cell images (a), linescans of GFP-anillin intensity (b), and quantification of ACE frequency (c) in HT-1080 cells expressing GFP-anillin migrating in different PDMS environments. Scale bar: 10 μm. **(d)** Presence of endogenous ACEs in wildtype HT-1080 and MDA-MB-231 plated on 2D substrates (Scale bars: 50 μm), as shown by immunofluorescence staining and anillin colocalization on the plasma membrane with RhoA and actin (inset; Scale bars: 10 μm). Linescanning of anillin, RhoA and actin showing their colocalization on the plasma membrane. **(e)** Representative Western blot showing varying expression levels of anillin and RhoA in different cell types. **(f)** Anillin nuclear exit occurs either concurrently with or after NE ruptures. Both G1/S and S-synchronized cells form ACEs at their trailing edge during cell entry into confinement before the first sign of NE rupture. ACEs are further enriched after rupture occurs. Timelapse taken at 108 sec intervals. Yellow dashes indicate microchannel entrance. White numbers indicate time from start of channel entry (hr:min:sec). **(g)** Frequency of ACE formation at the rear and/or front of synchronized cells during their entry into confining channels and frequency of ACE formation before, at or after NE rupture (NER) events in synchronized cells (n≥68 cells from 2 experiments). **(h)** Representative images of an S-phase cell experiencing repeated NE ruptures which resulted in intensifying ACEs. Scale bar: 10 μm. **(i)** Timing of anillin escape to the cytoplasm following the first NE rupture (n≥33 cells from 2 experiments). Statistical significance was assessed by one-way ANOVA followed by Tukey’s multiple comparisons (c) or two-way ANOVA followed by Dunnett’s post hoc test (g).

The assembly of the actomyosin-based contractile ring during cytokinesis critically depends on the activation of RhoA^24^ by the RhoGEF Ect2, which is anchored to the equatorial cortex via the scaffold protein anillin^23^. While anillin and Ect2 are predominantly localized to the nucleus during interphase^20, 21^, anillin can also be found at cell-cell junctions in epithelial cell monolayers^28^, suggesting that cell-type-specific mechanisms regulate its nuclear-cytoplasmic partitioning. Live cell imaging of HT1080 cells expressing GFP-anillin revealed its predominant nuclear localization accompanied by a variable degree of low cytoplasmic signal accumulating on the membrane when grown on 2D surfaces (Fig 2a,b). Both nuclear and cytoplasmic endogenous anillin was also observed by immunofluorescence in 5 additional tissue culture cell types, including MDA-MB-231 triple-negative breast cancer cells and A431 epidermoid carcinoma cells (Suppl. Fig. 2a,b).

While most HT-1080 cells migrating in moderately-confining channels exhibited a similar nuclear-cytoplasmic localization pattern as cells in 2D, higher degrees of confinement (30 and 9 μm^2^) sharply increased the frequency of cells with anillin concentrating at cell edges within narrow bar- or arch-like areas >2–3μm in length (called ACEs; Fig. 2a–c and Suppl. Fig. 2c), as determined by live cell imaging and immunofluorescence (Suppl. Fig. 2c–f). Similar localization patterns were also detected with MDA-MB-231 and human osteosarcoma (HOS) cells in moderately-confining and confining channels (Suppl. Fig. 2g,h).

As previously reported^24, 28^, anillin colocalizes with filamentous actin and RhoA at these cytoplasmic sites of accumulation (Fig. 2d) irrespective of varying anillin and RhoA protein expression levels (Fig. 2e). Moreover, co-immunoprecipitation experiments showed that the constitutively active form of RhoA (RhoA Q63L) readily interacts with anillin in cytoplasmic extracts of HT1080 cells synchronized in S-phase (Suppl. Fig 2i), demonstrating that the anillin-RhoA scaffolding function is not limited to cytokinesis.

Considering the cell cycle-mediated changes in nuclear volume^29^ and anillin protein levels^30^, we next used time-lapse imaging to examine the spatial distribution of GFP-anillin at distinct cell cycle stages in confined migration. To this end, we prepared populations of HT-1080 cells expressing GFP-anillin and nuclear localization signal (NLS)-mCherry that were enriched in early G1/S transition versus mid-S-phase by synchronization via serum withdrawal or serum withdrawal and hydroxyurea treatment, respectively^31^. Confocal live cell imaging revealed that entry into confinement for both G1/S and S phase cells was frequently marked by the accumulation of GFP-anillin within ACEs predominantly at the trailing cell edges (Fig. 2f,g). Although the formation of these spatially localized GFP-anillin-rich zones was dynamic over time and varied from cell to cell, several prevailing patterns were observed: irrespective of cell cycle stage, ACEs were primarily induced by cell entry, forming either before, during or shortly after nuclear entry into confining microchannels (Suppl. Fig. 2j and **Suppl. Video 1**). In most cells, ACEs formed either before or in the absence of NE rupture (Fig. 2g), as determined by concurrent monitoring of nuclear-to-cytoplasmic NLS-mCherry ratio (Fig. 2f,h). Although the frequency of cells with at least one nuclear rupture was similar in G1/S and S-synchronized cells (Suppl. Fig. 2k), S-phase cells experienced the first NE rupture at an earlier timepoint and underwent NE ruptures at a higher rate compared to G1/S cells in confinement (Suppl. Fig. 2l,m), presumably due to their larger nuclear size and more extreme nuclear stretching in confinement (Suppl. Fig. 2n,o), as quantified by nuclear morphometric analysis of maximum intensity projections.

We next examined GFP-anillin nucleocytoplasmic relocalization during confinement-induced NE rupture identified by the abrupt decrease of nuclear-to-cytoplasmic NLS-mCherry ratio (Fig. 2h). Quantification of the subcellular distribution of GFP-anillin in G1/S- or S-phase synchronized cells revealed that while the release of nuclear GFP-anillin to the cytoplasm coincided with NE rupture in ~40% confined cells, it was delayed by up to 20 min in the rest of the cells (Fig. 2i), suggesting that nuclear retention of anillin slows its cytoplasmic escape upon the breach of NE integrity. Of note, live-cell imaging showed that NE rupture promoted anillin localization to cell edges concurrently with a decrease in nuclear-to-cytoplasmic GFP-anillin ratio for both synchronized (Fig. 2f,h and **Suppl. Video 2**) and unsynchronized cells (Suppl. Fig. 2p,q), suggesting that anillin released from nucleus preferentially accumulated within the ACEs. At the same time, while NLS-mCherry rapidly re-accumulated in the nucleus upon the exit from mitosis (t=0–120 min), the nuclear re-accumulation of anillin (t≥300 min) was delayed (Suppl. Fig. 2r), suggesting that cytoplasmic anillin retention competes with its active nuclear import. Together, these data indicate that binding partners of anillin within nucleus and cytoplasm hinder its free exchange between the two compartments. Moreover, cytoplasmic anillin in cells at different phases of the interphase is recruited to the cell poles during nuclear entry into confining microchannels, and is further enriched as a consequence of confinement-induced NE rupture.

### Anillin localization is regulated by the stiffness and pore size of the local microenvironment *in vitro* and *in vivo*

**Figure 3.**
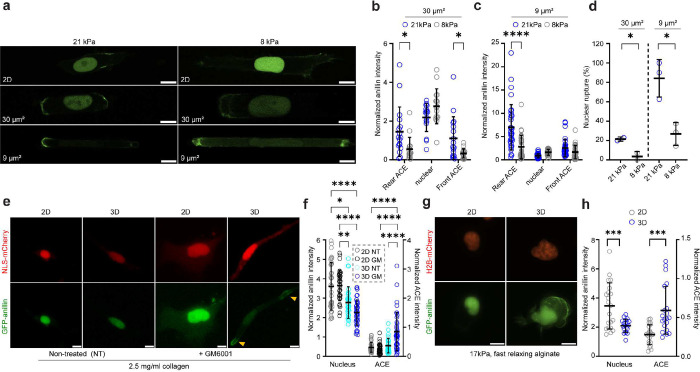
Anillin is enriched in the cytoplasm and recruited to ACEs during migration in compliant hydrogel-based channels, 3D collagen and viscoelastic alginate gels. **(a)** Representative images of GFP-anillin localization in HT-1080 cells on 2D and inside 30 μm^2^ and 9 μm^2^ compliant channels of 21 or 8 kPa stiffness. Scale bars: 10 μm. **(b,c)** Quantification of GFP-anillin intensity in the rear, nuclear, and front cell regions of cells inside compliant (21 or 8 kPa) channels with cross-sectional area of 30 μm^2^ (b) (n≥16 cells from 2 experiments) or 9 μm^2^ (c) (n≥32 cells from 4 experiments). **(d)** NE rupture frequency in cells inside compliant (21 vs 8 kPa) 30 μm^2^ and 9 μm^2^ channels (n≥7 cells per experiment from 3 experiments). **(e)** Representative images of GFP-anillin and NLS-mCherry localization in HT-1080 cells on 2D or 3D collagen gels with or without the MMP inhibitor GM6001. Arrowheads indicate GFP-anillin at the cell poles. Scale bars: 10 μm. **(f)** Quantification of GFP-anillin intensity in the nucleus and within ACEs on 2D or 3D collagen gels with or without GM6001 (n≥31 cells from 5 experiments). **(g)** Representative images of GFP-anillin and H2B-mCherry localization in HT-1080 cells on 2D or in 3D viscoelastic alginate gels. Scale bars: 10 μm. **(h)** Quantification of GFP-anillin intensity in the nucleus or within ACEs 2D or in 3D alginate gels. (n≥20 cells from 2 experiments). Statistical significance was assessed by two-way ANOVA followed by Sidak’s posthoc test (b,d) or unpaired t-test (c,h) or Kruskal-Wallis followed by Dunn’s (f (nucleus)) or one-way ANOVA followed by Tukey’s after log transformation (f (cell periphery)).

We sought to extend our findings from stiff PDMS-based confining microchannels to other physiologically relevant microenvironments. First, we examined the subcellular distribution of anillin in a hydrogel encapsulated microchannel array (HEMICA), which allows for the precise control of channel stiffness and size^32^. The HEMICA device consisted of an array of 4-walled, compliant polyacrylamide (PA)-based channels, which were either confining (*A*~30 μm^2^, *W*~10 μm, *H*~3 μm) or tightly confining (*A*~9 μm^2^, *W,H*~3 μm) to recapitulate the dimensions of the PDMS device. The microchannels were fabricated with stiffnesses of either 8 or 21 kPa to emulate (patho)physiologically-relevant conditions^33^. In line with data using PDMS channels, anillin was primarily localized in the nucleus of cells on 2D surfaces irrespective of substrate stiffness (Fig. 3a) but became increasingly polarized, forming ACEs as the stiffness and degree of confinement increased (Fig. 3b,c). Notably, cells inside stiffer (21 kPa) displayed a higher accumulation of anillin at both cell front and rear compared to cells inside softer (8 kPa) confining channels (Fig. 3b), whereas in tight confinement a more intense anillin signal was only detected at the cell rear (Fig. 3c). The higher anillin accumulation in migrating cells inside stiffer than softer channels is presumably attributed to the higher rate of nuclear rupture (Fig. 3d).

Next, we examined anillin localization in a 3D ECM environment, which recapitulates the stiffness and porosity of *in vivo* tissues^34^. Pronounced nuclear rupture has previously been observed in cells embedded in 3D collagen-I gels following matrix metalloproteinase (MMP) inhibition due to the inability of cells to widen pores via enzymatic cleavage of collagen fibers^16^. We verified this finding using NLS-mCherry-tagged cells embedded in 2.5 mg/mL collagen-I gels (Fig. 3e). Given that nuclear rupture amplifies the formation of ACEs in confining channels (Suppl. Fig. 2p,q), we predicted increased cytoplasmic anillin accumulation in 3D collagen gels especially after MMP inhibition. Indeed, while GFP-anillin was mainly nuclear on 2D collagen-I gels with some presence in the cell periphery similar to cells on 2D glass (Fig. 3e,f, Suppl. Fig. 3a), GFP-marked ACEs- were >2-fold higher in MMP-inhibited cells migrating through 3D gels (Fig. 3e,f). Of note, in the absence of MMP inhibition, the nuclear intensity of GFP-anillin was significantly lower in 3D than on 2D gels, suggesting that the moderately increased NE rupture frequency in 3D (Suppl. Fig. 3b) likely contributed to the decrease in nuclear anillin levels.

While collagen gels recapitulate some aspects of the physiological tissue microenvironment, natural ECM and living tissues exhibit various viscoelastic behaviors, often displaying stress relaxation over different characteristic time-scales (t_1/2_)^35^. To extend our findings to matrices of physiologically relevant viscoelastic properties, we examined anillin localization in fast relaxing (t_1/2_=1 min) alginate gels with a stiffness of 17 kPa. GFP-anillin was primarily localized to the nucleus of cells on 2D gels (Fig. 3g). In contrast, the nuclear localization of anillin decreased significantly in 3D gels concomitant with a ~2-fold increase in ACE intensity (Fig. 3g,h).

**Figure 4.**
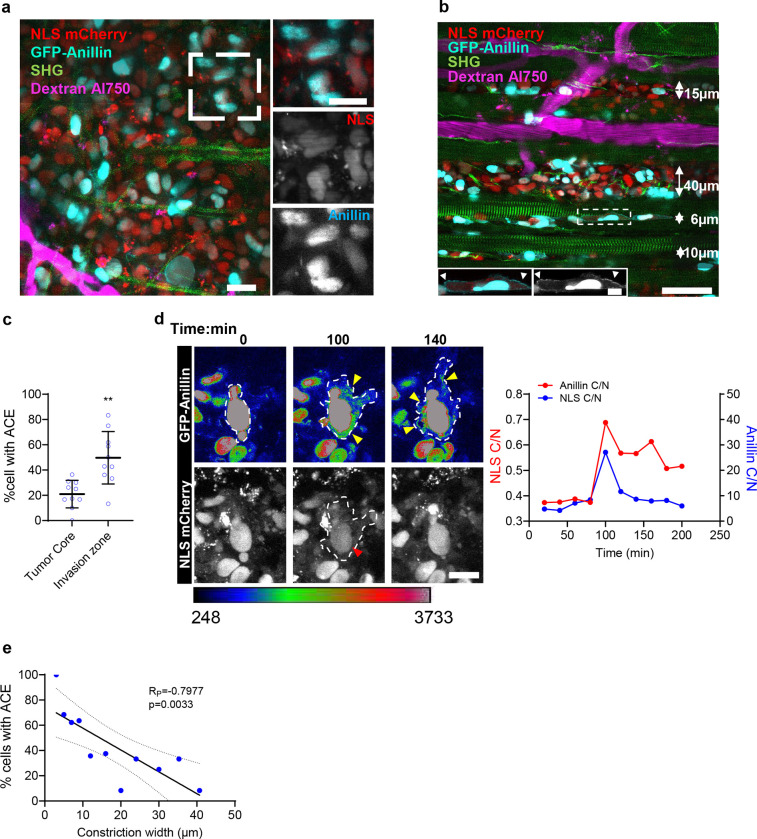
Confinement enhances anillin nuclear exit and accumulation on plasma membrane in vivo. **(a)** HT-1080 GFP-anillin/NLS-mCherry tumor xenografts invading the mouse dermis were monitored by intravital multiphoton microscopy through a dorsal skin-fold chamber. Images represent overview and details of the tumor core obtained 2 days after tumor implantation 50 μm below the tumor surface. NLS-mCherry (red), GFP-anillin (cyan), second harmonic generation (SHG) positive collagen fibers (green), AlexaFluor750 positive macrophages (magenta). Scale bars: 20 μm. **(b)** Single-cell and multicellular invasion along interstitial clefts between blood vessels, myofibers and interstitial collagen networks of different width. Double-headed arrows indicate the average width of each tissue track after entry of tumor cells. Example cell in the zoomed images show GFP-anillin (cyan or grayscale) redistributed to the cell edge (arrowheads in zoomed insets) migrating between two myofibers. Scale bar: 50 μm (overview), 5 μm (detail). **(c)** Percentage of cells with cytoplasmic anillin in the invasion zone versus the tumor core. Data represent mean percentage per region from a total of 204 cells from 9 regions in the tumor core (206 cells) from 3 mice. **(d)** Onset of NLS-mCherry and GFP-anillin exit from the nucleus and localization to the cytosol. Left panels, time-lapse recording and single-slice display of example cell localized near the tumor edge (related to **Suppl. Video 3**). Yellow arrowheads, cytoplasmic anillin; red arrowhead, transient decrease of NLS intensity in the nucleus. Scale bar: 20μm. Right graph, time-dependent ratio of cytoplasmic versus nuclear intensity (C/N) of both signals. **(e)** Inverse correlation between the percentage of cells with cytoplasmic anillin and the width of interstitial space. Data represent n=204 cells obtained from 30 tracks pooled from 3 mice. Values represent the mean±SD. **p<0.01 relative to tumor core by unpaired t-test (c); Pearson correlation (e).

Finally, we examined the localization pattern of anillin in cells migrating *in vivo*. HT-1080 tumors expressing GFP-anillin and NLS-mCherry were implanted into the deep dermis of nude mice bearing an optical imaging window for *in vivo* monitoring in real-time^36^. Tumor cell invasion into the collagen-rich interstitial tissue was longitudinally monitored 4 to 11 days post-implantation; 3D tissue constituents, including fibrillar collagen and myofibers (SHG), macrophages and blood vessels (70 kD Dextran-Alexa Fluor 750), were co-recorded together with the translocation of GFP-anillin from the nucleus into the cytoplasm. Whereas the majority of non-invading cells at the tumor boundary retained primarily nuclear anillin localization (Fig. 4a), translocation of GFP-anillin to the plasma membrane was increased in invading cells supporting a pro-metastatic role for anillin *in vivo* (Fig. 4b,c). Initiation of NLS-mCherry leakage correlated with anillin enrichment to the cell cytoplasm and plasma membrane (Fig. 4d and **Suppl. Video 3**). While this enrichment was observed in conjunction with repeated NLS-mCherry leakage events (Suppl. Fig. 3c), it could also occur independently of nuclear rupture (Suppl. Fig. 3d), presumably due to the cytoplasmic redistribution of anillin, as noted *in vitro*. Consistent with a mechanoresponse, the percentage of cells displaying cytoplasmic anillin localization increased with increasing confinement *in vivo*, reaching almost 100% in narrow perimuscular tissue clefts (Fig. 4e). Taken together, these data suggest that both substrate stiffness and pore size regulate the subcellular distribution of anillin *in vitro* and *in vivo*.

### Anillin and Ect2 promote RhoA/myosin II-dependent contractility and bleb-based migration

**Figure 5.**
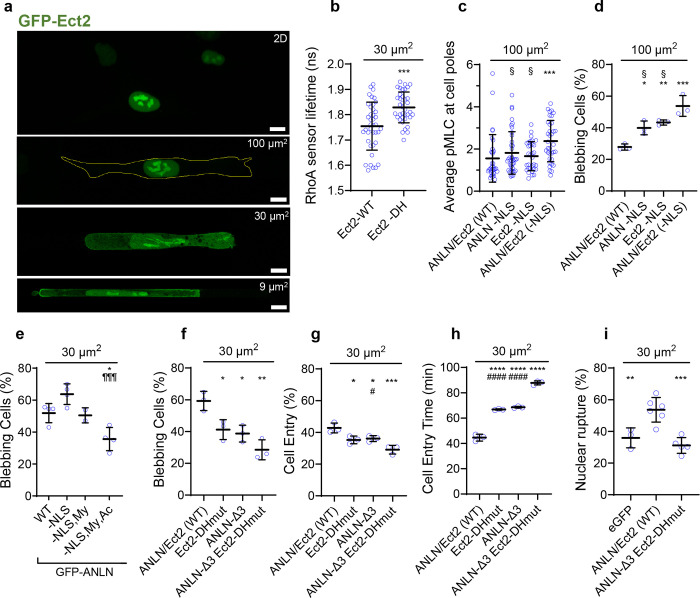
Cytoplasmic enrichment of anillin and Ect2 mediated by NE rupture promotes RhoA/myosin II-dependent contractility and efficient cell entry into confinement. **(a)** Representative images of HT-1080 cells showing GFP-Ect2 nuclear exit upon increasing confinement. Scale bars: 10 μm. **(b)** Donor fluorescence lifetime of RhoA activity biosensor RhoA2G for HT-1080 cells ectopically expressing HA-Ect2 or HA-Ect2 with mutations in its DH domain in confining channels, as measured by FLIM-FRET (n≥37 cells from 3 experiments). **(c)** pMLC intensity at the cell poles, as assessed from fixed and stained HT-1080 cells ectopically expressing either both GFP-anillin and HA-Ect2 (ANLN/Ect2 (WT)), or GFP-anillin or HA-Ect2 with a mutated NLS domain (ANLN-NLS or Ect2-NLS), or both GFP-anillin and HA-Ect2 with mutated NLS domains in moderately-confining channels (n≥32 cells from 3 experiments). **(d)** Percentage of blebbing cells in moderately-confining channels for HT-1080 cells ectopically expressing the constructs in (c) (n≥47 cells per experiment from 3 experiments). **(e)** Percentage of blebbing cells in confining channels for HT-1080 cells ectopically expressing GFP-anillin (WT), GFP-anillin with deletion in its NLS domain (-NLS), or NLS and myosin binding domains (-NLS,My), or NLS, myosin and actin binding domains (-NLS,My,Ac) (n≥10 cells per experiment from ≥2 experiments). **(f)** Percentage of blebbing cells in confining channels for HT-1080 expressing both GFP-anillin/HA-Ect2 (ANLN/Ect2 (WT)), or HA-Ect2-DHmut, or GFP-anillin with NLS, Myosin, Actin deletion (ANLN-Δ3), or both GFP-anillin-Δ3 and HA-Ect2-DHmut (n≥42 cells per experiment from 3 experiments). **(g,h)** Percentage of cells that entered into confining channels (g) and cell entry time (h) for HT-1080 cells expressing the constructs in (f) (n=30 cells per experiment from 3 experiments). **(i)** Frequency of nuclear rupture in HT-1080 cells expressing GFP-anillin/HA-Ect2 (ANLN/Ect2 (WT)) or GFP-anillin-Δ3 and HA-Ect2-DHmut during migration in confining channels. HT-1080 cells expressing eGFP were used controls (n≥33 cells per experiment from ≥3 experiments). Values represent mean±S.D. *p<0.05, **p<0.01, ***p<0.001, ****p<0.0001 relative to Ect2-WT, or anillin (WT)/Ect2 (WT) or GFP-anillin (WT); ¶¶¶p<0.001 relative to anillin lacking NLS; §p<0.05, §§p<0.01, §§§p<0.001 relative to anillin/Ect2 (-NLS); #p<0.05, ####p<0.0001 relative to ANLN-Δ3 and Ect2-DHmut as assessed by unpaired t-test (b), one-way ANOVA followed by Tukey’s multiple comparisons test (d-i) after log-transformation (c).

Considering the roles of anillin and the RhoGEF Ect2 in activating RhoA at the contractile ring during cytokinesis^22, 24^, we hypothesized that the presence of these proteins in the cytoplasm may contribute to elevated RhoA/myosin II-dependent contractility. First, we examined how confinement alters the subcellular distribution of Ect2. GFP-Ect2 did not seem to display an inherent cytoplasmic pool but instead resided almost exclusively in the nucleus of cells on 2D and in moderately-confining channels (Fig. 5a). As NE rupture became more frequent with increasing degree of confinement (Suppl. Fig. 1d), Ect2 became increasingly cytoplasmic. However, unlike anillin, which formed prominent ACEs at the cell poles (Figs. 2a, 3a), Ect2 was mostly diffuse throughout the cytoplasm and, at the same time, accumulated uniformly at the plasma membrane (Fig 5a). To test its contribution to RhoA/myosin II-dependent contractility, we expressed different Ect2 mutant constructs in HT-1080 cells. Ectopic expression of Ect2 with mutations in its DH catalytic domain (Suppl. Fig. 4a), which is required for nucleotide exchange on RhoA^37^, reduced RhoA activity in confining channels relative to wild-type Ect2 as quantified by increased donor fluorescence lifetimes via FLIM (Fig. 5b). Overexpression of Ect2 that harbors mutations in its NLS regions^37^ (Suppl. Fig. 4a), which sequester Ect2 in the cytoplasm (Suppl. Fig. 4b), increased cell contractility, as evidenced by immunostaining against phosphorylated myosin light chain (pMLC) and elevated fluorescence intensity in cell cytoplasm (Suppl. Fig. 5a). Of note, no elevated pMLC was detected at the cell poles (Fig. 5c), which is consistent with the diffuse distribution of Ect2 following its escape to the cell cytoplasm (Fig. 5a, Suppl. Fig. 4b). Consistent with the notion that elevated contractility promotes bleb formation, overexpression of Ect2 with mutated NLS increased cell blebbing in both moderately-confining and confining channels (Fig. 5d, Suppl. Fig 5c). Conversely, Ect2 knockdown (Suppl. Fig. 4c) suppressed RhoA activity and the extent of cell blebbing in confining channels (Suppl. Fig. 5b,c). Similarly, impairing Ect2 activity via mutations in its DH catalytic domain reduced cell blebbing in confinement (Suppl. Fig. 5c).

To assess the individual and potentially cooperative roles of anillin and Ect2 in confined migration, we ectopically expressed different mutant constructs of anillin and Ect2 in HT-1080 cells (Suppl. Fig. 4d,g). Deletion of the NLS domain sequestered GFP-anillin in the cytoplasm (Suppl. Fig. 4e,f) and increased myosin II contractility and cell blebbing in moderately-confining channels (Suppl. Fig. 5a, Fig. 5d). Interestingly, ectopic expression of both anillin and Ect2 NLS mutants led to even more pronounced increases in cell blebbing and myosin II contractility with markedly intense pMLC signals at the cell poles (Fig. 5c,d, Suppl. Fig. 5a,d). These mutants emulate the spatial distribution of RhoA/ROCK/myosin II-dependent contractility in confinement (Fig. 1c). Collectively, these data suggest that cytoplasmic accumulation of anillin and Ect2 activates actomyosin contractility at the cell poles, and promotes the conversion of cells from a mesenchymal to a blebbing phenotype.

We next sought to elucidate how anillin and Ect2 mediate their effects. We hypothesized that anillin, following confinement-induced cytoplasmic re-distribution and nuclear exit, acts as a scaffold protein that brings together its binding partners RhoA, myosin, actin, and Ect2 (Suppl. Fig. 4d)^23, 38^, where Ect2 further activates RhoA. To test this, we compared the phenotypes of HT-1080 cells overexpressing full-length anillin (WT) to anillin lacking an NLS domain as well as other deletions that prevent anillin from interacting with its critical binding partners: myosin binding domain deletion (-NLS, -My), or actin and myosin binding domain deletions (-NLS, -My, -Ac) (Suppl. Fig. 4d). Although removal of the NLS domain localized anillin primarily to the cell poles (Suppl. Fig. 4f, 5d), it did not significantly alter the percentage of cells with a blebbing phenotype in confinement (Fig. 5e), presumably because a significant fraction of endogenous anillin already exists in the cytoplasm of confined cells. Deletion of the NLS and myosin binding domains of anillin (-NLS, -My) led to the presence of anillin at the cell poles and throughout the cytoplasm (Suppl. Fig. 4f) without significantly affecting the percentage of blebbing cells (Fig. 5e). In line with this finding, myosin IIA or IIB depletion did not alter the percentage of cells with ACEs on 2D surfaces, suggesting that myosin II may be dispensable for anchoring anillin to the membrane (Suppl. Fig. 5e). In distinct contrast, removing NLS, actin and myosin binding domains (-NLS, -My, -Ac) resulted in a punctate distribution of anillin throughout the cytoplasm (Suppl. Fig. 4f), revealing the pivotal role of actin in anchoring anillin to the cell poles, leading to ACE formation. Moreover, this triple deletion markedly reduced the percentage of blebbing cells compared to anillin lacking only its NLS domain in confinement (Fig. 5e). These findings with the triple deletion mutant are also in accord with the attenuation of cytoplasmic pMLC levels and polarization at the cell edges observed in moderately-confining channels relative to anillin lacking only NLS (Suppl. Fig. 5d,f,g).

Because RhoA/myosin II contractility promoted cell entry into confining microenvironments (Fig. 1g,h) and nuclear rupture^15^, we examined the roles of anillin and Ect2 in these processes. Ectopic expression of constructs that disrupts either anillin polarization via -NLS,-My,-Ac deletion or Ect2 activity via DH mutations suppressed the percentage of blebbing cells (Fig. 5f) and delayed their entry into stiff confining channels (Fig. 5g,h) and compliant tightly-confining channels (Suppl. Fig. 5h). Overexpression of both mutants resulted in an additive inhibitory effect (Fig. 5f–h), and also reduced the percentage of cells displaying NE rupture as well as the frequency of nuclear rupture events in confinement (Fig. 5i and Suppl. Fig. 5i). To extend the physiological relevance of our findings, we employed the 3D spheroid model which mimics aspects of cell dissociation from a primary tumor^39, 40^. In line with observations in stiff and compliant microchannels, HT-1080 cells expressing the dual anillin and Ect2 mutant exhibited reduced dissociation from spheroids (Suppl. Fig. 5j). To validate our findings with other cancer cell lines, we demonstrate that the MDA-MB-231 cells expressing the dual mutant also displayed a reduced invasive potential as evidenced by a delayed cell entry time in stiff confining channels and markedly decreased cell dissemination from 3D spheroids (Suppl. Fig. 5k,l). Taken together, these data suggest a positive feedback regulation between actomyosin and anillin: in confinement, cytoplasmic anillin is redistributed to the cell poles primarily via actin, and together with Ect2 locally increases RhoA/myosin II contractility. Increased actomyosin contractility promotes NE rupture, which exacerbates nuclear egress of cytokinesis proteins anillin and Ect2, and facilitates cell entry into confining channels and bleb-based migration.

Prior studies demonstrated the role of calcium-dependent signaling in elevation of contractility in confinement^41, 42^. Specifically, moderate compression of the nuclear envelope along the dorsoventral axis increased nuclear and endoplasmic reticulum membrane stretching, resulting in release of calcium from internal membrane stores^41, 42^. Intracellular calcium, intracellular stretch-activated calcium channels and the nuclear tension sensor cPLA2 were required for inducing contractility in moderate confinement (*H*=5 μm)^41, 42^. However, inhibition of this pathway via treatment with BAPTA-AM or 2-APB or the cPLA2 inhibitor pyrrophenone had no effect on cell blebbing or cell entry in confined (*H*=3 μm) channels (Suppl. Fig. 5m,n), suggesting that the effects of confinement-induced nuclear rupture have a more dominant role in cell phenotype regulation.

### Anillin and Ect2 promote tumor cell invasion and extravasation *in vivo*

**Figure 6.**
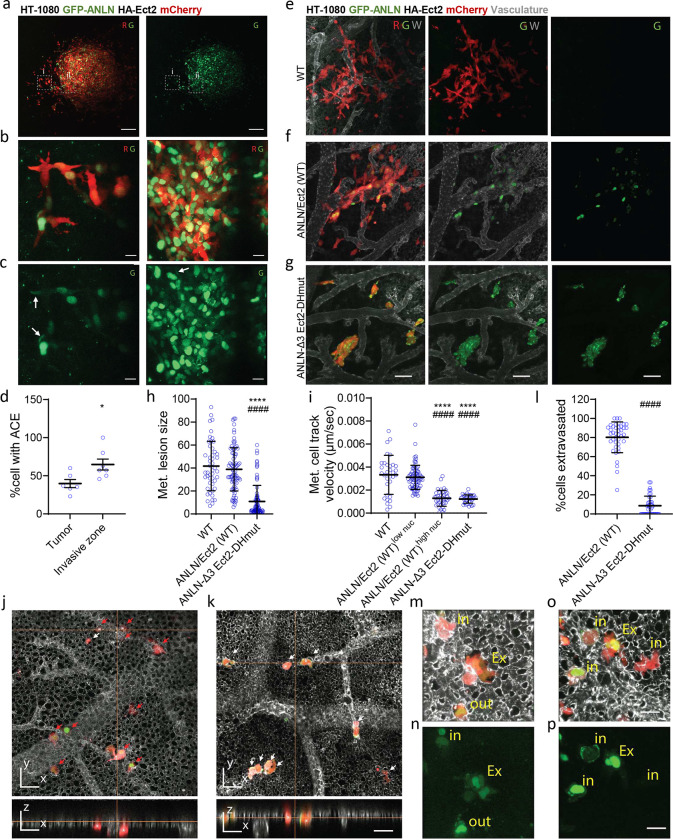
Dual anillin and Ect2 mutation suppresses cell invasion in ex ovo chick embryo cancer xenograft model. **(a)** Representative images of mCherry-tagged HT-1080 cells expressing GFP-anillin (WT)/HA-Ect2 (WT) during invasion into the surrounding tissue (i) from the primary tumor mass (ii). Scale bar: 100 μm. **(b,c)** Composite image (b) and GFP-ANLN localization (c) of cells in the invasive zone from inset (i) (left) or primary tumor mass from inset (ii) (right). White arrows indicate ACEs. Scale bar: 10 μm. **(d)** Quantification of ACE frequency in cells in the tumor core versus invasive zone (n≥77 cells from 6 tumors from 8 animals). **(e-g)** Representative images showing metastatic lesions formed by mCherry-tagged HT-1080 WT cells (e), mCherry-tagged GFP-anillin (WT)/HA-Ect2 (WT) (dual WT) cells (f) or GFP-anillin-Δ3 and HA-Ect2-DHmut (dual mutant) (g) with maximum intensity projection views. Scale bar: 100 μm. **(h)** Quantification of average number of cancer cells in metastatic lesions formed by WT, dual WT or dual mutant HT-1080 cells (n≥48 lesions from 15 animals). **(i)** Quantification of the average track velocity of metastatic HT-1080 WT cells, or dual WT cells with low or high nuclear anillin, or dual mutant cells. **(j,k)** Representative images showing HT-1080 dual WT cells (j) or dual mutant cells (k) extravasating out of the CAM vasculature via 3D reconstructions with maximum intensity projection views. Red arrows point to cells that have extravasated; white arrows point to the cells that are still inside the vasculature. Upper panels show xy views, lower panels show xz views. Scale bar: 20 μm. **(l)** Quantification of average percentage of cells that extravasated for the cells shown in j and k (n≥20 cells per animal from 20 animals). (**m-p)** Representative, high magnification images showing HT-1080 dual WT cells that are in the process of extravasating out of the CAM vasculature via maximum intensity projection views. Lower panels show GFP (anillin) channel only. in=intravascular cell; ex=extravasating cell; out=extravasated cell. Exposure in (o) and (p) are increased to show cytoplasmic anillin. Scale bar: 20 μm. Values represent mean±S.D. *p<0.05, ****p<0.0001 relative to tumor or WT; ####p<0.0001 relative to dual WT or dual WT^low nuc^ as assessed by unpaired t-test (d), Kruskal-Wallis followed by Dunn’s multiple comparisons (h,i), or Mann-Whitney test (l).

To study the roles of anillin and Ect2 in cancer cell invasion *in vivo*, we employed an ex ovo chick embryo cancer xenograft model^43–45^. mCherry-labeled HT-1080 cells co-expressing wild type GFP-anillin and HA-Ect2 (ANLN/Ect2 (WT)) or anillin and Ect2 dual mutant (ANLN-Δ3 Ect2-DHmut, Suppl. Fig. 4g) were injected between the chick embryo chorioallantoic membrane (CAM) ectoderm and endoderm layers, and their invasion was monitored 5 days post tumor cell inoculation using intravital imaging. High magnification imaging of dual wildtype GFP-anillin and HA-Ect2 expressing cells (Fig. 6a–c and Suppl. Fig. 6a) reveal that in agreement with data obtained from mouse IVM (Fig. 4), a higher proportion of cells that invaded out of the primary tumor displayed anillin accumulation at the cell edges compared to those remaining inside the tumor core (Fig. 6d). Disruption of both anillin and Ect2 function had a profound effect on HT1080 cancer cell invasion, resulting in a minimal number of invasive cells at the primary tumor front (Suppl. Fig. 6b–d).

To assess the contributions of anillin and Ect2 in the formation of metastatic colonies, HT1080 cells expressing dual wild type or dual mutants of anillin and Ect2 were injected intravenously into the CAM vasculature^43^ and allowed to form metastatic colonies for 4 days. While wild type-anillin and Ect2 HT-1080 cells formed rapidly growing invasive metastatic lesions, comparable to wildtype HT1080 mCherry control cells (Fig. 6e,f,h), the anillin and Ect2 dual mutants formed compact noninvasive lesions that were smaller in size (Fig. 6g,h and **Suppl. Video 4**). This intervention also resulted in a significantly decreased cancer cell track velocity (Fig. 6i).

Cancer cell extravasation requires directional extension of invadopodia that are necessary for forced vascular wall breaching^3^. We thus tested if disruption of both anillin and Ect2 function is detrimental for successful cancer cell extravasation. Indeed, the anillin and Ect2 dual mutant cells extravasated poorly relative to wild type cells (Fig. 6l–k). High magnification visualization of dual wild type cells also revealed an increase in cytoplasmic signal of anillin, which was observed inside the vasculature during extravasation and persisted even after the extravasation process was complete (Fig. 6m–p). These data suggest that spatial confinement imposed on cells during extravasation *in vivo* as well as physical cues present in the vasculature, such as fluid shear stress, regulate the re-distribution of anillin and Ect2 resulting in RhoA activation, which is critical for efficient cell invasion and extravasation.

## DISCUSSION

Besides the localization of anillin and the RhoA activator Ect2 predominantly in the cell nucleus, and their canonical role in regulating cell division^21, 24, 38^, it is clear that the scaffolding function of anillin in activating RhoA is not limited to mitosis^28, 46^ and is likely cell type-specific. We herein delineated a novel role for these cytokinesis proteins in the regulation of migration, invasion and extravasation. We found that physical confinement promotes cytoplasmic re-distribution and accumulation of anillin at the poles of migrating cells prior to NE rupture, which is further enhanced when ruptures induce anillin and Ect2 egress to the cytoplasm. The elevated cytoplasmic localization of anillin -and its recruitment to the plasma membrane- was detected in stiff and compliant confining channels, 3D collagen gels and 3D viscoelastic alginate gels *in vitro.* Increasing substrate stiffness and reducing channel size promoted anillin cytoplasmic exit and polarized accumulation at plasma membrane. Cytoplasmic anillin binds to actin and myosin at the cell poles, and works additively with Ect2 to locally activate RhoA/myosin II-dependent contractility and facilitate cell entry into confining spaces. Consistent with *in vitro* studies, cytoplasmic accumulation of anillin was elevated during invasion *in vivo*, particularly in tissue regions with high confinement (<10 μm track width) in mice. Importantly, cells with plasma membrane-proximate anillin were more likely to invade efficiently out of the primary tumor in a chick embryo model. Cells overexpressing the inactive forms of anilin and Ect2 displayed a markedly reduced invasive potential *in vitro* and extravasation in the chick embryo model, suggesting the anillin-Ect2-dependent RhoA activation might be required for the initiation of the metastatic process. The dual mutations presumably inactivate RhoA via dominant negative sequestration of the endogenous proteins.

The confinement-induced translocation of anillin to the cytoplasm was detected across a range of stiffnesses and pore/channel sizes *in vitro* and *in vivo*. Upon decreasing the channel stiffness, a higher degree of confinement is required to induce anillin nuclear exit. This is presumably due to the lower myosin-II dependent contractility on softer substrates^47^, which is required to promote NE rupture^15^ and anillin/Ect2 escape as well as ACE formation via actomyosin binding. Likewise, narrow (<10 μm) tissue clefts *in vivo* discourage multicellular arrangement of collective invasion, but instead enforce chain-like single-cell migration, with bilateral cell contact to comparably stiff structures of the dermis, including collagen bundles and myofibers (5 to >100 kPa)^48, 49^. Conversely, in wider channels, invading cells are less confined, as they interact with both tissue structures and much softer bodies of neighbor cells (<2 kPa)^50^. As such, the frequency of cytoplasmic anillin localization is elevated for cells in single strands confined between the perimysium and myofibers as compared to cell clusters invading through wider channels between myofibers. Thus, confinement and stiffness of heterogenous cell and tissue topographies *in vivo* likely generate cooperating mechanochemical triggers in inducing and sustaining anillin translocation to the membrane and support cytoskeletal contractility.

Prior work identified a key role for intracellular calcium signaling in cell blebbing in confinement^41, 42^, which was not detected in our study. This difference may be attributed to the distinct confining geometries in these studies. Specifically, cells were unconfined laterally and moderately confined vertically compared to our microchannels (*H*≥5 μm^41, 42^ versus 3 μm height), which may explain why NE rupture events were not observed in these prior studies^41, 42^. Moreover, calcium signaling is activated as a result of nuclear compression, while the presence of cytoplasmic anillin seems to be constitutive across a range of migratory cell types. Its membrane enrichment at moments of cell entry into confinement, notably preceding NE rupture events in most cases, suggests that anillin becomes anchored by pre-polarized actin and myosin at the cell poles^13, 15^, and activates RhoA to further elevate contractility. Additionally, in highly-confining environments, damage to the nuclear membrane as a result of frequent rupture events may prevent the sustained elevation of nuclear membrane tension that is required to trigger calcium-dependent activation of contractility.

Instead, rupture-mediated escape of anillin and Ect2 to the cytoplasm appears to be the key factor leading to elevated contractility in highly confining environments. Ect2, although primarily diffuse in the cytoplasm upon nuclear exit, also localizes at the cell poles and locally activates the RhoA/myosin II pathway. Despite the additive effect of anillin and Ect2 on increasing cell contractility, previous work suggests that anillin and Ect2 are strong binding partners, forming a complex to promote microtubule stabilization during cytokinesis^23^. Thus, the possibility of a direct interaction between these molecules at the cell poles can not be excluded. Also, Ect2 can be recruited to caveolae in response to low membrane tension^14^. It is likely that anillin and Ect2 act as part of a positive feedback loop in which their cytoplasmic accumulation increases RhoA/myosin II contractility, thereby facilitating additional NE rupture events^15, 16, 51^ and exacerbating anillin and Ect2 exit.

The high frequency of NE rupture events and the partial nuclear envelope sealing in confinement contributes, at least in part, to their sustained cytoplasmic localization. NE rupture events were detected approximately once per hour during migration in confining channels^15^, and nuclear rupture repair proceeded over the course of ~100 min^16, 17^, which can explain the incomplete repair observed in our system. Upon their cytoplasmic translocation, the import of any proteins, including anillin and Ect2, back to the nucleus may be delayed by their association with cytoplasmic constituents. Cell type- or cell state- (such as cell cycle-stage-) specific cytoplasmic partners could then be responsible for the diversity of the cytoplasmic versus nuclear pool of such “predominantly nuclear” proteins. Also, we cannot exclude a role for nuclear pore complexes (NPCs) in anillin and/or Ect2 nuclear cytoplasmic trafficing. Our data revealed that the pool of the RhoA scaffold anillin in the cytoplasm has major consequences for the RhoA activation at the poles of migrating cells in confinement. This anchoring is mediated by anillin’s actin and myosin binding domains, likely acting in concert with the dynamic anillin association with phosphoinositide-4,5-P_2_ (PIP_2_) at the plasma membrane^28^. Acting redundantly and supporting each other, these scaffolding mechanisms may facilitate the cortical persistence of the anillin/Ect2 machinery beyond individual NE rupture events.

The cytoplasmic accumulation of anillin and Ect2 is accompanied by a bleb-based migration phenotype, which was particularly evident during migration in confining microchannels and 3D viscoelastic hydrogels. Cell blebbing was also observed in moderately-confining microenvironments primarily for cells expressing anillin and Ect2 in which their NLS sites were mutated, which triggered their cytoplasmic localization. However, in mouse dermis the frequency of membrane blebbing was not as pronounced even though anillin was still detected in the cytoplasm, albeit at lower levels. This might be attributed to elevated levels of Rac activators in the tumor microenvironment *in vivo* due to cytokine release, which may lead to the co-existence of cortical contractility and filamentous protrusions.

In summary, we propose a model by which confinement induces cytoplasmic re-distribution of cytokinesis proteins and their enrichment via NE rupture. Anillin becomes anchored by its binding partner actin at the cell poles and is recruited to PIP2 at the plasma membrane via its PH domain^28^, whereas Ect2, although diffuse in the cytoplasm and uniformly accumulated at the plasma membrane, is also available for binding to anillin within ACEs at the cell poles. As such, anillin and Ect2 work together to hyperactivate cellular contractility and facilitate cell entry into confining channels. In line with the notion that Rho-ROCK and actomyosin contractility are drivers of metastasis^52^, we found that disruption of anillin and Ect2 function suppresses cancer cell invasion at the tumor fronts and extravasation. While this study focuses on anillin and Ect2, it is probable that NE rupture induces cytoplasmic translocation of many other nuclear proteins, as has already been reported for DNA repair factors^53^. The propensity of migrating cells to lose their nuclear compartmental identity in confinement might contribute to the deregulation of cytoplasmic processes controlling cell migration and other behaviors. Overall, our work reveals that confinement regulates the redistribution and enrichment of anillin and Ect2 in the cytoplasm, which alters intracellular signaling and promotes cell invasion and extravasation.

## Online Methods

### Experimental Methods

#### Cell culture and pharmacological inhibitors

Human HT-1080 fibrosarcoma cells (kindly provided by Dr. Wirtz at Johns Hopkins), MDA-MB-231 breast cancer cells, osteosarcoma (HOS) cells, A431 epidermoid carcinoma, and human foreskin fibroblasts (HFF-1) were cultured in Dulbecco’s Modified Eagle Medium (DMEM) containing 4.5 g/L glucose, L-glutamine, and sodium pyruvate (Gibco) and supplemented with 10% heat-inactivated fetal bovine serum (Gibco, 16140071) and 1% penicillin/streptomycin (10,000 U/mL, Gibco, 15140122). BRC-196 breast cancer cells (kindly provided by Dr. Seagel at McGill University) were cultured in DMEM/F-12 containing 3.151 g/L glucose, 15 mM HEPES, L-glutamine, and sodium pyruvate supplemented with 10% heat-inactivated fetal bovine serum (Gibco, 16140071), 10 nM β-estradiol (Sigma, E8875), 0.4 μg/ml hydrocortisone (MilliporeSigma, H0135), 5 ng/ml heregulin-β1 (STEMCELL Technologies, 78071) and 4 μg/ml insulin (Sigma, I9278). Cells were grown in an incubator maintained at 37°C and 5% CO_2_, and sub-cultured every 2–4 days.

In select experiments, cells were treated with the following pharmacological agents and corresponding vehicle controls. Reagents were obtained from Sigma Aldrich unless otherwise noted: Y27632 (Y0503, 10 μM), hydroxyurea (8 mM), blebbistatin (B0560, 50 μM), GM6001 (364206, 20 μM), 2-APB (Tocris Bioscience, 1224, 100 μM), BAPTA AM (Invitrogen, B6769, 25 μM), pyrrophenone (Cayman Chemical, 13294, 0.1 μM).

#### Cloning, lentivirus preparation, transduction, and transfection

To generate shRNA lentiviral vectors, the target sequences were subcloned into pLVTHM (Addgene, Cambridge, MA, plasmid # 12247) using MluI and ClaI as restriction sites or pLKO.1 (Addgene, Cambridge, MA, plasmid #8453) using AgeI and EcoRI as restriction sites. The target sequences are: Scramble Control: sh1 (GCACTACCAGAGCTAACTCAGATAGTACT), human MYH9 (ACGGAGATGGAGGACCTTATG), human MYH10 (GGATCGCTACTATTCAGGA).

WT GFP-Anillin and GFP-Anillin_^68^AAA^70^ plasmids were kindly provided by the Wilde Lab^20^ (University of Toronto, Toronto, Ontario, Canada). GFP-Ect2 was generously given by the Yap Lab^54^ (The University of Queensland, St. Lucia, Brisbane, Queensland, Australia). Anillin mutant constructs (GFP-anillin lacking NLS, NLS and myosin, NLS, actin, and myosin, NLS and AHD) were kindly provided by the Glotzer Lab^24^ (University of Chicago, Chicago, Illinois). pFugW-HA-Ect2 wildtype and mutants (NLS- and DH- mutant Ect2) were generously given by the Cox Lab^37^ (University of North Carolina at Chapel Hill, Chapel Hill, NC). The following plasmids were purchased from Addgene: pLenti.PGK.LifeAct-GFP.W (plasmid #51010), pLenti.PGK.H2B-mCherry (plasmid # 51007), pQC NLS mCherry IX (plasmid #37354), pEGFP-Anillin (plasmid #68027), tetO-FUW-eGFP-RHOA-Q63L (plasmid #73081), RhoA2G FRET biosensor (plasmid #40176, #40179), psPAX2 (plasmid #12260), and pMD2.G (plasmid #12259), VSV.G (plasmid #14888), pLV-EF1α-IRES-puro (plasmid #85132), pLV-EF1α-IRES-Hygro (plasmid #85134).

Transient transfections (Fig. 5e, and Suppl. Fig. 5c) as well as lentivirus production and infection were performed as described previously^13, 15^.

For siRNA knockdown, scramble (sc-37007) and Ect2 (sc-35259) siRNA were purchased from Santa Cruz Biotechnology, and CHMP2A siRNA from Dharmacon^16^. Cells were transiently transfected with siRNA using the Lipofectamine RNAiMax Kit (Invitrogen) according to the manufacturers protocol.

#### Photolithography and device fabrication

Polydimethylsiloxane (PDMS) microfluidic devices, consisting of an array of parallel channels with a fixed channel length of 200 μm and different heights (*H*) and widths (*W*), were fabricated as described previously^55–57^. Based on their cross-sectional areas, channels were classified as moderately-confining (*W*×*H*=10×10 μm^2^), confining (*W*×*H*=10×3 μm^2^) or tightly confining (*W*×*H*=3×3 μm^2^). For cell migration experiments, channels were coated with 20 μg/mL rat tail collagen I (Gibco #A1048301).

#### Microfluidic device seeding and live cell imaging

Cell seeding was performed as described previously^15^. Briefly, cells were detached (0.05% trypsin-EDTA (Gibco)), centrifuged (300g for 5 min) and resuspended in serum-free DMEM (1% penicillin/streptomycin) to a concentration of 5 × 10^6^ cells/mL. The cell suspension (10–20 μL) was then seeded into the microfluidic device via pressure-driven flow. Devices were incubated at 37°C, 5% CO_2_ while cells were adhering and spreading in the device. To create a chemotactic gradient, the bottom three wells of each device were filled with serum-free DMEM (1% penicillin/streptomycin) while the top well was filled with serum-containing DMEM (10% FBS, 1% penicillin/streptomycin).

Cells were imaged every 5–10 min for 4–12 h on an inverted Nikon Eclipse T*i* microscope (Nikon, Tokyo, Japan) with automated controls (NIS-Elements; Nikon) and a ×10/0.45 numerical aperture Ph1 objective using time-lapse microscopy. Cells were maintained on a temperature and CO_2_-controlled stage-top incubator (Okolab, Pozzuoli, Italy or Tokai Hit, Shizuoka-hen, Japan) during the entire course of the experiments. For select experiments, FITC and TRITC filters were used for fluorescent imaging.

#### Cell phenotype analysis

Cells were allowed to migrate in PDMS-based channels for 4–5 h at which timepoint cells were fixed and stained with Hoechst 33342 and phalloidin (as detailed below) and observed using a Nikon AXR confocal with 40x water objective or an inverted Nikon Eclipse T*i* microscope (Nikon, Tokyo, Japan) using a 40x air objective. Migration phenotype was manually tabulated using the criteria described in^13, 15^.

To calculate the percentage of cell entry, we measured the total number of cells within a distance of 50 μm from channel entrances and quantified the fraction of these cells that fully entered into microchannels. Cell entry time was defined as the duration between cell protrusions first extending into the interior of the microchannels and full cell entry.

#### Actin staining, immunofluorescence imaging and quantification

For actin staining related to cell blebbing analysis, cells were fixed with 4% paraformaldehyde (PFA) (Affymetrix, Inc.), permeabilized in 0.1% Triton X-100 (Sigma), and blocked in 1% bovine serum albumin (Sigma). Cells were stained with rhodamine or Alexa Fluor 488 phalloidin (1:100, Invitrogen #R415 or A12379) and Hoechst (1:2500, Invitrogen).

For IF in Fig. 2b and Supp. Fig. 2a, cells were grown in 96-well glass-bottom plates (Cellvis, P96–1.5H-N) and fixed with 4% PFA (Electron Microscopy Sciences 157–14-S) in PBS, pH 7.4, for 10–15 min at room temperature (RT). Samples were permeabilized and DNA-stained (1% Triton X-100 (Sigma #P7949), 0.05% Tween-20 (Sigma), Hoechst 33342 10 μg/ml in TBS pH 7.4; 3–5 min at RT), incubated with blocking buffer (10% normal donkey serum (Millipore-Sigma S30–100ml) in PBS, pH 7.4, 0.05% NaN_3_ (Sigma S2002)) and incubated with primary antibodies (rabbit anti-anillin (Sigma-Aldrich, HPA005680, 1:200), mouse anti-RhoA (Santa Cruz Biotechnology, sc-418, 1:100) diluted in blocking buffer (overnight at 4°). After incubation with secondary antibodies and phalloidin (Alexa Fluor 488 donkey anti-rabbit (Invitrogen A32790n, 1:1000), Alexa Fluor 555 Donkey anti-mouse (Invitrogen A32773, 1:1000), Alexa Fluor 647 Phalloidin (Invitrogen A22297, 1:400)) and washes, the plates imaged with ImageExpress Micro Confocal high content microscope (Molecular Devices), with 20x air objective in spinning disc confocal mode with 60 μm pinhole. A 3×3 grid of images per each well contained 300–2500 individual cell images/well. MetaXpress software was used to analyze the background-corrected mean cytoplasmic to nuclear ratios (C/N) in raw, unaltered images, using the translocation-enhanced module (Molecular Devices). Data in Supp. Fig. 2b are from 3 independent biological replicates (one with two technical replicates/plates).

ImageJ was used to uniformly subtract image specific out-of-cell fluorescence for each channel before assembling representative IF images (Fig. 2d, Suppl. Fig. 2a) with Adobe Photoshop 2023. Linescan analyses (10 μm-wide) in Fig 2a,b were prepared with ImageJ and colored with GraphPad Prism 10.

For immunofluorescence in the remaining figures, cells were fixed with 4% PFA (Affymetrix, Inc.), permeabilized in 0.1% or 0.2% Triton X-100, and blocked in 5% BSA (Sigma, A7030)/0.05% Tween-20/0.05% NaN_3_ or 5% Bovine Serum Albumin (BSA) (Sigma)/2% Normal Goat Serum (Cell Signaling Technology 5425S)/0.2% Triton X-100. Samples were incubated overnight at 4°C with the following primary antibodies: anillin (Sigma-Aldrich, HPA005680, 1:100)), HA (Cell Signaling, C29F4, 1:600), or phospho-myosin light chain 2 (Cell Signaling, 3671S, 1:50) antibody. Afterwards, samples were washed 3x with PBS and incubated with the following secondary antibodies: Goat Anti-Rabbit IgG H&L, Alexa Fluor 488 (ThermoFisher, A11034, 1:200), Goat Anti-Rabbit IgG H&L, Alexa Fluor 568 (ThermoFisher, A11011, 1:200), Goat Anti-Mouse IgG H&L, Alexa Fluor 488 (ThermoFisher, A11001, 1:200), and Goat Anti-Mouse IgG H&L, Alexa Fluor 568, (ThermoFisher, A21043, 1:200). All antibodies were prepared in blocking buffer. Nuclei were also stained with Hoechst (1:4000–1:2500, Invitrogen) and actin with Alexa Fluor 488 or rhodamine phalloidin when applicable.

#### Fluorescence imaging and quantification

All fluorescence data, except for those in Fig. 2d and Supp. Fig 2a, were acquired on a Nikon A1 or AXR confocal microscopes (Nikon, Tokyo, Japan) using a 63X oil objective with a 1.4 numerical aperture or 40x water objective with a 1.15 numerical aperture, respectively. 640-nm, 567-nm, 488-nm, and 405-nm lasers were used for imaging. Fluorescence intensity quantification was performed using ImageJ. For anillin, the front and rear plasma membrane and the nucleus were selected as depicted in Suppl. Fig. 2d. Fluorescence intensity was measured in selected regions and normalized to total cell fluorescence intensity. In 3D collagen and viscoelastic alginate gels, the cell pole with the greatest fluorescence signal was quantified and identified as the cell periphery.

For pMLC quantification, fluorescence intensity for whole cell, nucleus, and front and rear plasma membrane were quantified. Cytoplasmic pMLC intensity is the mean fluorescence of the cytoplasmic area excluding the nucleus (Suppl. Fig. 2d). Mean fluorescence of the poles is normalized to mean cytoplasmic intensity excluding the nucleus and the poles.

#### Cell cycle synchronization

For G1/S-phase synchronization, cells were serum-starved in 0.25% FBS for 48 h, then released with full media (DMEM containing 10% FBS and 1% P/S) 30 min before the experiment^31^. For S-phase synchronization, cells were serum-starved in 0.25% FBS for 48 h, released with full media for 5 h, then treated with 8 mM hydroxyurea for 3 h before the experiment^31^.

#### ACE quantification

Cell z-scans were taken at 0.5-μm intervals and the z-plane with strongest membrane anillin signal was chosen. GFP-anillin intensity was analyzed along a 4-pixel-wide linescan running between the membrane region displaying anillin signal and the cytoplasm. Cells on 2D, 3D and moderately-confining channels are rendered positive for ACE if they have ≥5 μm of membrane region with anillin intensity at least twice that of the surrounding cytoplasmic region. Similarly, in confining channels, ACE-positive cells are considered those with at least twice the anillin intensity signal at the cell poles relative to the surrounding cytoplasmic region.

#### Nuclear rupture imaging and quantification

cells expressing NLS-mCherry were imaged on a Nikon A1 confocal microscope (Nikon, Tokyo, Japan) using a Plan Apo 20x air objective with a 0.75 numerical aperture and a resolution of 1024×512 pixels. A central z-plane of cells inside confining channel or z-stacks at 0.5-μm interval were acquired. For quantification of nuclear ruptures, the reduction of nuclear NLS-mCherry signal with the corresponding increase in its cytoplasmic intensity is considered to be a rupture event. Conversely, the recovery of nuclear signal and reduction of cytoplasmic signal is marked as a NE repair event. Cells were analyzed from when their nuclei reached the channel entrances until the cell protrusions reach channel exit. Cells whose nuclei were obstructed by particles or cellular debris during confined migration were excluded.

The movies of migrating cells in confinement were manually inspected to identify the x/y positions of the centers of nuclei in the first frame corresponding to full nuclear entry into microchannels. The nuclei images were first segmented using a custom program developed in MATLAB^58, 59^. The area (A), perimeter (P), long axis length and short axis length of the segmented nuclei were then computed using MALTAB image processing toolbox. The aspect ratio was calculated as the ratio of the long axis length and short axis length and circularity is 4πA/P^2^.

Manual linescan analyses with ImageJ were used to identify movie frames indicating NE rupture events as signaled by the abrupt increase of cytoplasmic and decrease of nuclear NLS-mCherry signal. In parallel, a 10-pixel wide line scan with ImageJ in the GFP-anillin channel was used to identify movie frames corresponding to the formation of front or rear ACEs. Time stamps of all changes in nuclear/cytoplasmic NLS-mCherry and ACE detection in the GFP-anillin channel were recorded in Excel sheets and used for the calculation of the frequency of NE ruptures and timing of front and rear ACEs with respect to the NE rupture. Similarly, line scans of the nuclear and diffuse perinuclear cytoplasmic GFP-anillin with ImageJ were used to detect the timing of nuclear GFP-anillin exit.

#### FLIM of RhoA FRET sensors

Confocal FLIM of live cells that were stably expressing the RhoA2G sensor was performed as described previously^15^ using a Zeiss LSM 780 microscope and a PicoQuant system consisting of the PicoHarp 300 time-correlated single photon counting (TCSPC) module, two hybrid PMA-04 detectors, and Sepia II laser control module.

#### FLIM reconvolution, image segmentation, and segmentation quantification

The FLIM data was processed as described previously^15^ using SymPhoTime 64 (PicoQuant) software.

#### Western blotting

To perform Western Blots in different cell lines (Fig. 2e and Supp Fig. 2a), sub-confluent cell cultures were harvested by trypsinization. Following trypsin deactivation with FBS, cell pellets were washed 2X in D-PBS (Gibco, 14190–144) before resuspension in 4% SDS, 120 mM Tris/HCl, pH 6.8, followed by heating at 100°C for 5 min. The lysates were sonicated (Branson Sonifier 250, power 4, 5s), protein concentration measured with Micro BCA Protein Assay Kit (Thermo Pierce, 23235) and samples stored at −80°C or used immediately for western blotting. Before electrophoresis, samples were reconstituted at 1 μg/μl in reducing 2x SDS Laemmli Sample buffer (Thermo, J61337.AC) and heated at 100°C for 3 min. Samples (10 μg lysate/well) were separated on 4–20% SDS PAGE Criterion gels and blotted to PVDF membrane with TransBlot Turbo (BioRad). After 30–60 min blocking with 5% Blotting Grade Blocker (BioRad, 1706404) prepared in TTBS (0.05% Tween 20, TBS, pH 7.4), blots were prepared as follows: the anillin/RhoA blots (Fig. 2d) were incubated with primary antibodies diluted in TTBS overnight at 4°C (rabbit anti-anillin (Sigma-Aldrich, HPA005680, 1:1000 combined with mouse anti-RhoA (Santa Cruz Biotechnology, sc-418, 1:200) and ECL (Thermo, SuperSignal West Femto, 34095) images developed after incubating with rabbit antibodies (HRP-linked anti rabbit, Cell Signaling Technology, 7074, 1:5000, 20 min). After inhibiting the HRP with 30% H_2_O_2_ (Millipore Sigma, H1009, 30 min at RT), the blots were developed with mouse antibodies (HRP-linked anti mouse, Cell Signaling Technology 7076, 1:5000, 20 min). The blots were stained with 25% Isopropanol, 10% Acetic Acid, 0.05% Coomassie R 250, washed with water, dried and imaged to verify equal total protein abundance between samples.

Western Blots for Supp. Fig. 4c were performed as previously described^56, 60^ using NuPage 3–8% or 4–12% gels and the following antibodies: Primary antibodies: mouse anti-Ect2 (Santa Cruz, sc-514750, 1:100), rabbit GAPDH (Cell Signaling Technology, 2118, 1:1000) was used as loading control. Secondary antibodies: Anti-mouse IgG, HRP-linked Antibody (Cell Signaling Technology, 7076S, 1:2000), anti-rabbit IgG, HRP-linked antibody (Cell Signaling Technology, 7074S; 1:2000).

#### Anillin co-immunoprecipitation with active RhoA.

HT1080 cell line stably expressing GFP-RhoA Q639L from tetO-FUW-eGFP-RHOA-Q63L were grown in two 15-cm culture dishes until 70–80% confluency. The plates were washed twice with DPBS and the growth medium (DMEM, 10% FBS) replaced with DMEM, 0.1% FBS. After 60–72 hours of serum starvation-induced G1/S arrest, cells were returned to DMEM, 10% FBS for 4 hours to initiate entry to S phase, before harvesting by trypsinization.

A modification of the nuclear/cytoplasmic fraction preparation^61^ was applied to obtain nuclear fraction-free cytoplasmic extracts, using digitonin permeabilization instead of Igepal C-630 (NP-40) to permeabilize plasma membranes. The cells were washed by centrifugation (1300 rpm, 2 min, RT) with D-PBS, resuspended in 15 ml 10 mM sodium phosphate, pH 7.4, proteinase inhibitor cocktail, EDTA-free (PIC, Roche 59813300), kept on ice for 10 min to induce hypo-osmotic swelling, pelleted at 1300 rpm, 4 min, RT and supplemented with 2 mM MgCl_2_, 5 mM GTP (pH 7.4). 40 mg/ml stock of digitonin (Sigma, D141) in DMSO was added in 50 μg/ml increments (final concentration) to reach >60–80% release of nuclei, as judged by bright field microscopy, typically requiring 200 μg/ml. A 30 μl aliquot of the permeabilized cell suspension was lysed with 1% NP-40 (final concentration, total cell extract, TCEx) and the rest loaded in 200–300 μl aliquots on top of 1ml sucrose cushions (10 mM Tris pH 7.4, 150 mM NaCl, 24% sucrose, PIC) in 1.7 ml Eppendorf tubes and centrifuged at 1500 g, 8 min, 4°C. The pellet (nuclei) was rinsed with D-PBS, PIC, lysed in 1% NP-40, D-PBS, pH 7.4, PIC, resulting in nuclear extract, NEx). The supernatant (cytoplasmic extract, CEx) was clarified of all remaining nuclei by three spins at 1500 g, 2 min, 4°C. Next, the clarified CEx was combined with pelleted and DPBS-washed 50 μl GFP-trap (Proteintech/Chromotech; gtma-20) beads or 50 μl binding control magnetic agarose (Proteintech/Chromotech; bmab, non-specific binding control). After incubation on a rotator at 4°C for 1.5 h, the beads were collected on a magnetic stand, supernatant removed, the beads washed 4X with 800 μl ice-cold TTBS, 0.05% NP40, transferred to new Eppendorf tubes and washed 2X. After removing the washing buffer, the beads were collected in 15 μl 2x SDS Laemmli sample buffer and heated for 5 min at 100°C. The protein concentration in TCEx, NEx and CEx was measured with BioRad Protein Assay (BioRad, 50000006). Aliquots of the equal protein amounts of the TCEx, NEx and CEx, along with the total of control- or GFP-trap bead elutions were separated on 4–20% SDS PAGE Criterion gels (BioRad), blotted to PVDF and developed with rabbit anti-anillin anibodies as described above. A portion of the blots corresponding to ~5–25kDa proteins was subsequently developed with rabbit anti-histone H3 antibodies (Cell Signalling Techology, 4499, 1:2000) to verify the absence of nuclear proteins in the cytoplasmic fractions used for the IP.

#### HEMICA device preparation and seeding

An array of parallel channels with a fixed channel length of 200 μm and different heights (*H*) and widths (*W*) were fabricated as described previously via soft photolithography^57, 62^. Acrylamide (A) (Bio-Rad, 40%) and N,N-Methylene bisacrylamide (B) (Bio-Rad, 2%) were mixed with distilled water to final concentrations of 8% A/0.1% B and 8% A/0.6% B corresponding to PA gels of elastic moduli 8 and 21 kPa, respectively^32^. The solutions were then degassed for 25 min. 10% ammonium persulfate (Bio-Rad) and 0.4% TEMED (Bio-Rad) were used as initiators of polymerization. Glass coverslips (Fisher Scientific, 22×40 mm) were activated as previously described^32^. The final polyacrylamide solutions were placed on top of the coverslip or wafer mold and flattened with an additional non-activated coverslip. After 50 min of polymerization, the gels were peeled off of the surfaces and submerged in PBS−/−. The gels were allowed to swell between 1 and 3 days in PBS−/− at 37°C ^32^. The stiffness of hydrogels was measured with an atomic force microscope.

Inlet and outlet holes were punched, the gels were lightly air-blown and sandwiched using 50 mg/mL bis(sulfosuccinimidyl)suberate (BS3, ThermoFisher Scientific,) as an adhesive^32^. Custom-made 60 mm petri dishes with an opening in the center were created and HEMICAs were glued on them in order to present an exposed glass center surface for imaging. Subsequently, HEMICA was submerged in PBS −/− for 10 min to rehydrate. After removal of the PBS, the microchannels surfaces were treated with 0.5 mg/mL sulfosuccinimidyl 6-(4’-azido-2’-nitrophenylamino)hexanoate (Sulfo-SANPAH, Thermo Fisher Scientific) as described in^32^. HEMICA was then submerged in 20 μg/mL collagen type I (Collagen I Rat Protein, Tail, Thermo Fisher Scientific) overnight at 37°C for functionalization^32^. After an overnight incubation, channels were washed with PBS −/− before seeding cells.

#### Collagen gel preparation and imaging

In select experiments, rat tail collagen I (Corning) was diluted in 0.1% acetic acid to form a 7.1 mg/mL stock collagen solution. To form collagen gels with embedded cells, appropriate volumes of collagen, 1x M199 Medium (Thermo Fisher), 10x M199 Medium, and cells were mixed on ice to create a solution with final concentrations of 2.5 mg/mL collagen I, 1x M199, and 2 × 10^5^ cells/mL. 200 mM NaOH was added incrementally while mixing on ice until a pH of 7.5 was obtained. In other experiments, rat tail collagen I (Corning #354236) was diluted to 2.5 mg/ml using 10x low-glucose DMEM (Sigma #D2429) and 1x complete DMEM media, and neutralized with 10 M NaOH on ice.

60 μL of this solution were pipetted into select wells of a glass-bottomed 96-well plate and incubated for 20 min at 37°C to allow gel formation, then 200 μL of DMEM (1% penicillin/streptomycin, 10% FBS) with or without GM6001 (EMD Millipore, 20 μM) were added to each well. To form collagen gels with cells on the surface, the above gel formulation procedure was repeated omitting cells, and 40 μL of this solution were pipetted into wells of a glass 96-well plate. After the 20 min gelation, 200 μL DMEM (1% penicillin/streptomycin, 10% FBS) containing 5 × 10^4^ cells/mL with or without GM6001 were added to the gel surface. After two days, media for all conditions was replaced, likewise with or without GM6001. On the third day, cells were imaged using a Zeiss LSM 800 confocal microscope using a 20X air objective and a resolution of 1024×1024 pixels. 567-nm and 488-nm lasers were used for imaging. Images were processed using Zen Blue software and ImageJ.

#### Cell dissociation from collagen gel-embedded spheroid

To form spheroids, 2,000 HT-1080 or 3,000 MDA-MB-231 cells were resuspended in ice-cold 150 μl of 2% v/v Matrigel (Corning #356230) in complete media (DMEM containing 10% FBS), added into round bottom ultra-low attachment 96-well plate (Corning, 7007), centrifuged for 5 min at 300 g, and then incubated at 37°C and 5% CO_2_ for 2 days. Collagen gel was formed by mixing 1 ml of rat tail collagen I (Corning, 354236) with 125 μl of 10x low-glucose DMEM (Sigma, D2429) and neutralized with ≥3.3 μl of 10 M NaOH on ice. To encapsulate spheroids in a collagen gel, 25 μl of gel was spread in a flat-bottom 24-well plate (Falcon, 3047) and polymerized for 40 min in a 37°C incubator to form a gel bed. Spheroids were collected with a 1000 μl pipet tip and gently resuspended and mixed with 120–130 μl of ice-cold collagen gel, then deposited onto the gel bed. Spheroid/gel mixture were allowed to polymerized for 1 h in a 37°C, 5% CO_2_ incubator, then covered with 1 ml of pre-warmed media and imaged on an inverted Nikon Eclipse T*i* microscope (Nikon, Tokyo, Japan) with automated controls (NIS-Elements; Nikon) and a ×10/0.45 numerical aperture Ph1 objective.

#### Quantification of cell dissociation from spheroids

Spheroids were visualized in ImageJ and the total number of dissociating cells was recorded up to the specified time.

#### Alginate gel preparation, fabrication, and mechanical testing

High molecular weight I1G alginate (~260 kDa) was purchased from KIMICA Corporation and was irradiated by cobalt-60 source to produce low MW alginate (~27 kDa). RGD-coupled alginate was prepared by coupling the peptide GGGGRGDSP (Peptide 2.0 Inc) using carbodiimide chemistry. Alginate was then purified by dialysis (3500 MWCO) against deionized water containing sodium chloride for 3 days, treated with activated charcoal, sterile filtered, lyophilized, and reconstituted in DMEM (1% penicillin/streptomycin, no FBS) following previously reported methods^35^. Calcium sulfate (CaSO4) was mixed with alginate as a source for the release of crosslinking calcium ions. The mixture was transferred to a glass plate coated with Sigmacote^®^, covered, and allowed to gel for 45 min. Gel disks, which were 15 mm in diameter and 2 mm thick, were equilibrated in DMEM (1% penicillin/streptomycin, no FBS) for 24h before mechanical testing. The elastic modulus and stress relaxation properties of alginate hydrogels were measured by compression tests of the gel disks using a MTS Criterion^®^ Series 40 Tensile Tester. The gel disks were compressed to 15% strain with a 2 mm/min deformation rate and a 100 Hz data acquisition rate. For the relaxation process, the compression strain was kept at 15%, as the load was recorded over time. The elastic modulus was derived from the slope of the linear region of the stress-strain curve (~5%-10% of strain). Stress relaxation properties were quantified by relaxation half-time (t_1/2_), which is the time for the initial stress to be relaxed to half its value during stress relaxation test.

#### 2D and 3D Alginate Gel Cell Seeding and Imaging

For 2D gel experiments, RGD-coupled alginate with low MW was reconstituted in DMEM (1% penicillin/streptomycin, no FBS) to 2.5% (w/v) and mixed with CaSO_4_ to form 17 kPa alginate hydrogels. The final Ca^2+^ concentration in the hydrogels was 68.3 mM. The final alginate hydrogels contained 2% (w/v) alginate. After gelling for 45 min, gels were punched to 15 mm disks and the thickness was controlled to 0.4 mm. Gels were transferred to a PDMS mold and customized holders were used to hold the gels at the bottom of the mold. 1 mL of DMEM (1% penicillin/streptomycin, 10% FBS) followed by 100 μL of cell suspension at a concentration of 2 × 10^4^ cells/mL were added to wells containing gels.

For 3D gel experiments, RGD-coupled alginate with low MW was reconstituted in DMEM (1% penicillin/streptomycin, no FBS) to 3% (w/v). First, alginate was mixed with cell suspension, then CaSO_4_ was added to form 17 kPa alginate hydrogel. The final Ca^2+^ concentration in hydrogels was 68.3 mM. The final alginate hydrogels contained 2% (w/v) alginate. Cell density in the alginate hydrogels was 10^6^ cells/mL.

For all gel conditions, cells were cultured at 37^◦^C, 5% CO_2_ and incubated overnight before imaging. Gels were imaged on a Nikon A1 confocal microscope (Nikon, Tokyo, Japan) using a 40X water objective with a 1.15 numerical aperture and a resolution of 1024×1024 pixels. 567-nm and 488-nm lasers were used for imaging.

#### Tumor Implantation and intravital multiphoton microscopy

Animal studies were approved by the Institutional Animal Care and Use Committee of The University of Texas, MD Anderson Cancer Center, which is accredited by the Association for Assessment and Accreditation of Laboratory Animal Care (IACUC protocols 00001002). Athymic nu/nu female mice were obtained from the Department of Experimental Radiation Oncology, M.D. Anderson Cancer Center. Dorsal skin-fold chambers were mounted on 8 to 12-week-old female athymic nu/nu mice as described previously^36^. In brief, the skin-fold chamber was mounted on a skin-flap on the back to cover the deep dermis after surgically removing the opposite side of the skin. One day post-surgery, pelleted HT1080 cells (2.5–5 × 10^5^ cells in 2–4 μl) stably expressing NLS-mCherry and GFP-anillin were injected into the dermis with a 30-G needle. Three tumors per chamber were implanted and monitored for up to 11 days. Intravital microscopy was performed on a LaVision TrimScope II scanner with three titanium-sapphire lasers (Ti:Sa, Chameleon-XR, Coherent) and two optical parametric oscillators compact systems (OPO, APE/Coherent, tunable excitation wavelengths range between 800 and 1300 nm) on days 2–11 to monitor tumor growth and subcellular distribution of NLS-mCherry and GFP-anillin. Therefore, mice were anesthetized with isoflurane (1–3% in oxygen), placed on a temperature-controlled stage (37°C) and the chamber was mounted on a holder. Blood vessels were visualized by retro-orbital injection of 70 kDa dextran (Invitrogen/ThermoFisher) labeled with AlexaFluor-750 (1 mg/mouse). Imaging was performed using an Olympus XLPLN25XWMP2 25X water objective (NA 1.05; 2 mm working distance). Sequential 3D stacks were acquired with three excitation wavelengths (880 nm, 1090 nm and 1280 nm) in two consecutive scans. Emission was detected using the following band pass filters: THG (1280 nm; ET450/60 nm); mCherry (1090 nm; ET595/40 nm), SHG (1090 nm; ET525/50 nm), Alexa Fluor 750 (1280 nm; ET810/90 nm), GFP (880nm; ET525/50 nm; Chroma Technology Inc.). 3D volumes were acquired for up to 250 μm penetration depth at a step size of 5 μm. Time-lapse recording with a frame interval of 20 min was performed for a maximum duration of 5 hours. 3D image stacks were reconstructed as maximum intensity projection, stitched and analyzed using NIH ImageJ.

#### *In vivo* mouse IVM image analysis

To perform the analysis of the cell signal intensity profiles over time, the ratio between the cytoplasmic and the nuclear (C/N) mean grey values were calculated in each frame for both the Anillin-GFP and the NLS-mCherry channels. The nuclear intensity values were obtained manually defining a region of interest in either nucleus or cytoplasm (NLS) or nucleus and cell edge (anillin). Intensity was calculated as the mean intensity, except membrane Anillin-GFP intensity, which was obtained as the sum of the lengths of the segments composing the membrane (manually segmented, line width of 4 pixels) multiplied by their average intensity.

To correlate the percentage of cells with cytoplasmic anillin with the geometry of the local invasion environment, the track width was quantified from the combined 3D SHG, fluorescent dextran and the cell-based fluorescence in orthogonal direction from the invasion direction and the associated the number of cells with ACEs for each invasion zone. ACE quantification was performed as described in the relevant section. This analysis was validated by independent, blinded analysis. The Manual Tracking (Cordelières F, Institut Curie, Orsay France) was used to analyze cell migration paths.

#### *Ex Ovo* Chick Embryo Cancer Xenograft Model

Fertilized White Leghorn chicken eggs, acquired from the University of Alberta Poultry Research Centre, were maintained in a humidified incubator at 38°C. After four days of incubation, embryos were removed from their shells and maintained under shell-less conditions in a covered dish at 38°C and 60% humidity as previously described^43–45^.

For the primary tumour or primary tumour invasive front imaging, day ten chicken embryos were injected with 1×10^5^ mCherry-labeled HT-1080 cells or mCherry-labeled HT-1080 cells expressing either wild-type GFP-anillin and HA-Ect2 (ANLN/Ect2 (WT)) or anillin and Ect2 dual mutant (ANLN-Δ3 Ect2-DHmut) in PBS directly in between CAM ectoderm and endoderm layers. Sterilized, circular coverslips (22mm diameter) were positioned on top of the tumour one day past tumour cell inoculation and image acquisition was performed three-four days later^42–44^.

For metastatic colony imaging, day ten chicken embryos were injected intravenously with 2.5×10^4^ mCherry-labeled HT-1080 cells or mCherry-labeled HT-1080 cells expressing either ANLN/Ect2 (WT) or ANLN-Δ3 Ect2-DHmut. Sterilized, circular coverslips (22mm diameter) were positioned on the CAM surface above metastatic colonies one day after tumour cell injection. Metastatic colonies were allowed to develop for four more days and single metastatic colonies were selected for visualization and analysis^42–44^.

In cancer cell extravasation experiments, 5×10^4^ mCherry-labeled HT-1080 cells expressing either ANLN/Ect2 (WT) or ANLN-Δ3 Ect2-DHmut were injected into the CAM vasculature. Cancer cell extravasation was analyzed 8h post injection as described in^63^.

#### Image Acquisition and Analysis

Real-time imaging of cancer cell invasion was performed by acquiring 4-dimensional image series of single cancer cells within the CAM tissue, as previously described^43–45^, using a Nikon A1r upright microscope (Nikon) fitted with a temperature regulated enclosure and a range of Nikon microscope objectives (10, 25x (WI) and 63x (oil)). Time 0 was defined as the time of the first image capture. 20 to 50 individual cells were tracked (for each cell line used in the experiments) using build in Volocity Object tracking module. Track velocity was calculated as average speed of the track. Track displacement rate (productivity) was calculated using build in Volocity module as total track displacement (straight line distance from the first track position to the last) divided by track time. For quantification of the cancer cell number per colony 25x z-stack images were acquired (2–5 μm step) and cancer cells were counted manually using Nikon Elements software. For quantification of invasive cancer cells at the primary tumors periphery individual (25x) z-stack images were analyzed using Nikon Elements software. For Anillin-GFP signal intensity quantifications primary tumor images (main tumor mass and invasive zone) were acquired at 60x magnification. All experiments were done in triplicates. All experimental data were plotted and analyzed for statistical significance using the Prism analysis module.

For quantification of anillin fluorescence intensity ratios 63x confocal images of main tumor mass and invasive zones were obtained. ImageJ was used to quantify anillin or mCherry fluorescence intensity within the nucleus or cytoplasm within the single optical slices (1μm). For time lapse tracking analysis image drift was corrected using the ImageJ Stack_Reg plugin (Biomedical Imaging Group, http://bigwww.epfl.ch/thevenaz/stackreg/).

ACE quantification was performed using ImageJ. For cells inside primary tumor masses, a 256×256 pixel ROI was placed in the middle of the tumor mass. All cells with at least half of their nuclei being inside the ROI were included in the analysis. All cells that were found in the invasive zones were analyzed. ACE quantification was performed as described in the relevant section above. Cells that had no mCherry signal or whose nuclear envelope were not distinguishable were excluded from our analysis.

#### Statistical analysis

Data represent the mean±S.D. or median with 95% CI from ≥3 independent experiments unless otherwise noted. The D’Agostino-Pearson or Shapiro-Wilk omnibus normality test was used to determine whether data are normally or log-normally distributed. Data sets with gaussian distributions were compared using Student’s t-test, one-way ANOVA test followed by a Tukey’s test for multiple comparisons, or two-way ANOVA test followed by a Sidak’s test for multiple comparisons. A Wilcoxon matched-pairs signed rank test was used to determine statistical significance. Log-normal data were transformed using Y=log(Y) formula before comparison. For non-Gaussian distributions, the nonparametric Mann-Whitney test was used comparing two conditions, while more than two groups were compared by Kruskal-Wallis test followed by Dunn's multiple comparison. Analysis was performed using GraphPad Prism 6, 7, 8, 9 or 10 Software. Statistical significance was identified as p<0.05. *p<0.05, **p<0.01, ***p<0.001 and ****p<0.0001.

## Supplementary Material

Supplement 1
