# Supplementary material for: Cytoplasmic accumulation and plasma membrane association of anillin and Ect2 promote confined migration and invasion
Source: Res Sq. 2024 Jan 10:rs.3.rs-3640969. Preprint. [Version 1] doi: 10.21203/rs.3.rs-3640969/v1 (PMC10802709; doi:10.21203/rs.3.rs-3640969/v1)
Supplement: Supplement 1 [file NIHPPrs3640969v1-supplement-1.pdf]

SUPPLEMENTAL VIDEOS

Supplemental Video 1. Cells displaying ACEs before their entry in confinement and enrichment after nuclear rupture. Representative time-lapse recording of an S-phase HT-1080 cell expressing GFP-anillin (WT) and NLS-mCherry entering and migrating inside a confining channel. Note the ACEs before cell entry and their enrichment following nuclear rupture. Exposure was uniformly increased to highlight ACEs before cell entry. Scale bars: 10µm.

Supplemental Video 2. Cells in confinement experiencing multiple nuclear ruptures. Representative time-lapse recording of an S-phase HT-1080 cell expressing GFP-anillin (WT) and NLS-mCherry inside a confining channel. Exposure was uniformly increased to highlight ACEs. Scale bar: 10 µm.

Supplemental Video 3. Initiation of NLS-mCherry leakage correlated with anillin enrichment to the cell cytoplasm and membrane *in vivo*. Representative time-lapse recording of an HT-1080 cell localized near the tumor edge. Onset of GFP-anillin (left, RGB rainbow LUT) and NLS-mCherry (right, grayscale) exit from the nucleus and localization to the cytosol. Yellow arrowheads, cytoplasmic anillin; red arrowhead, transient decrease of NLS intensity in the nucleus. Scale bar: 20 µm.

Supplemental Video 4. Disruption of RhoA-dependent contractility with GFP-anillin-Δ3 and HA-Ect2-DHmut leads to less invasive phenotype. mCherry-tagged HT-1080 cells expressing GFP-anillin-Δ3 and HA-Ect2-DHmut dual mutants formed more compact and less invasive lesions than GFP-anillin (WT)/HA-Ect2(WT) controls. Upper panels show mCherry and GFP channels; lower panels show GFP channel only. 20 min/frame; 7.6 h total duration; 10x magnification.

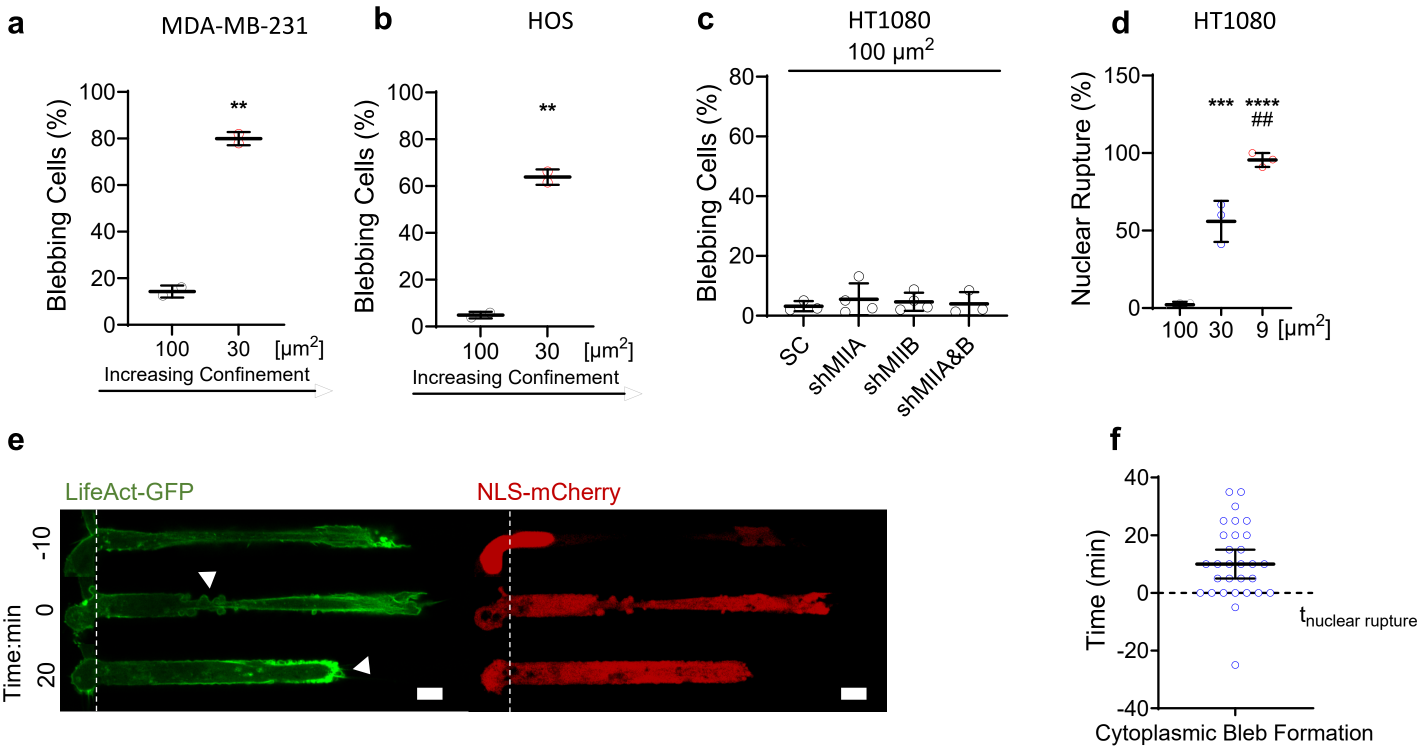

Supplemental Figure 1. Confinement induces nuclear rupture and cell blebbing. **(a, b)** Percentage of MDA-MB-231 (a) or HOS (b) cells migrating with a blebbing phenotype in moderately-confining (100 μm²) and confining (30 μm²) channels (n≥20 cells per experiment from 2 experiments). **(c)** Percentage of scramble control, MIIA-, MIIB-, or MIIA and IIB- knockdown HT-1080 cells migrating with a blebbing phenotype in 100 μm² channels (n≥15 cells per experiment from ≥3 experiments). **(d)** Percentage of HT-1080 cells that experienced NE rupture in 100 μm², 30 μm², and 9 μm² channels, as evaluated by NLS-mCherry exit into the cytoplasm (n≥10 cells per experiment from 3 experiments). **(e)** Image sequence of representative HT-1080 cells experiencing nuclear rupture, as assessed by NLS-mCherry localization, and subsequently forming cytoplasmic blebs, as assessed by LifeAct-GFP, in confining (30 μm²) channel. White dashed lines indicate channel entrance. White arrowheads indicate membrane blebs. Scale bar: 10 μm. **(f)** Time required for the initial formation of cytoplasmic blebs versus the first NE rupture in confining (30 μm²) channels, as assessed by cells expressing NLS-mCherry and LifeAct-GFP (n=30 cells from 3 experiments). Values represent mean±SD (a-d), or median with 95% CI (f). $^{**}p<0.01$, $^{***}p<0.001$, $^{****}p<0.0001$ relative to 100 μm², ##p<0.01 relative to 30 μm² as assessed by unpaired t-test (a,b) or one-way ANOVA followed by Tukey's multiple comparison (d).

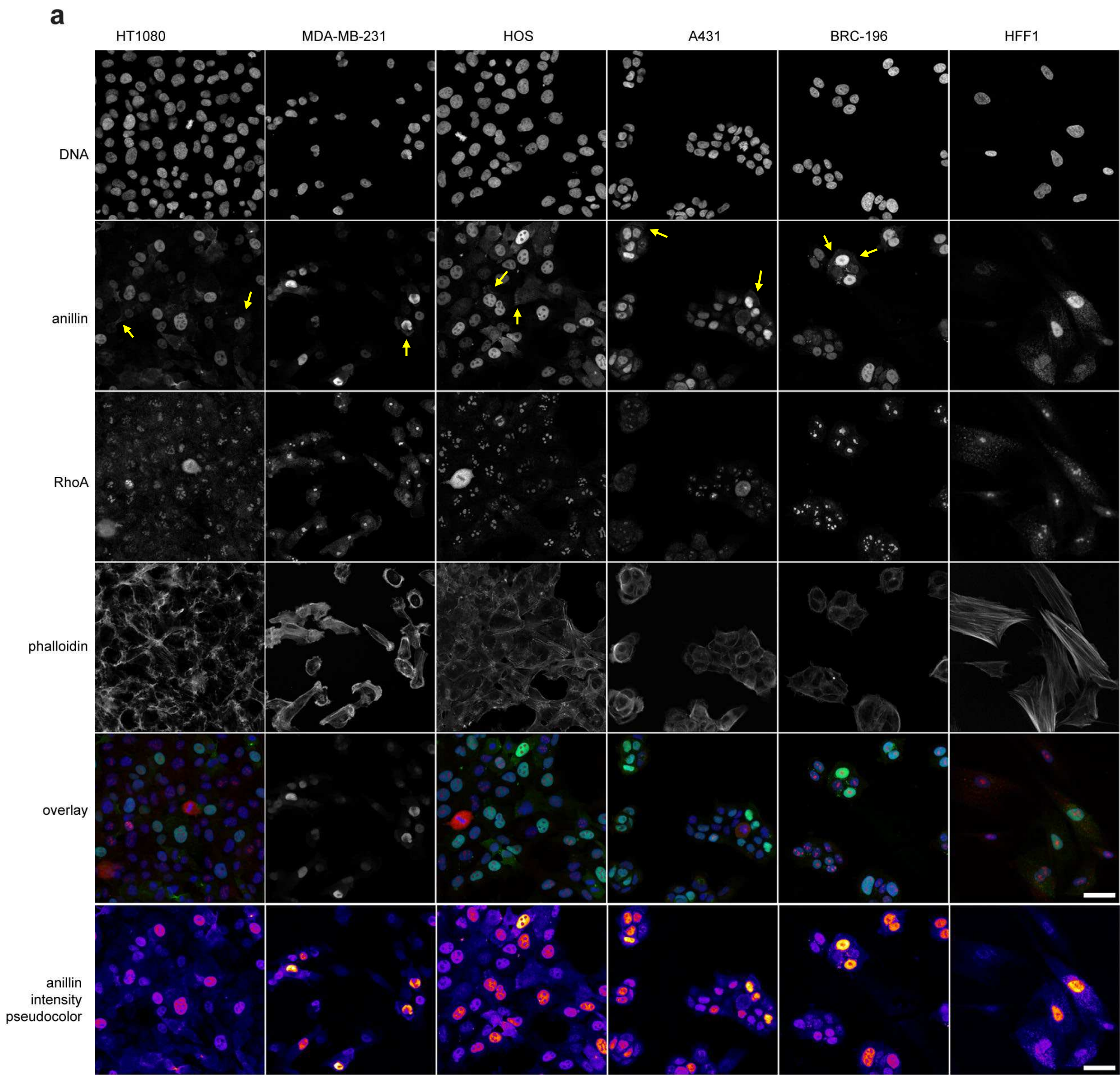

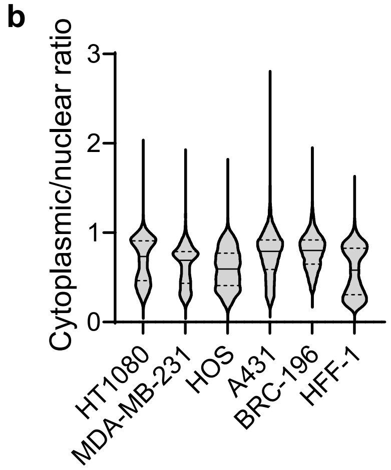

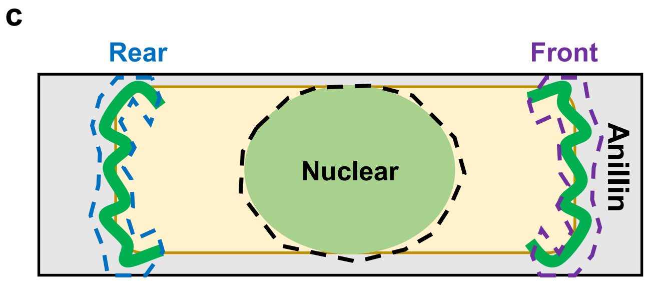

Supplemental Figure 2

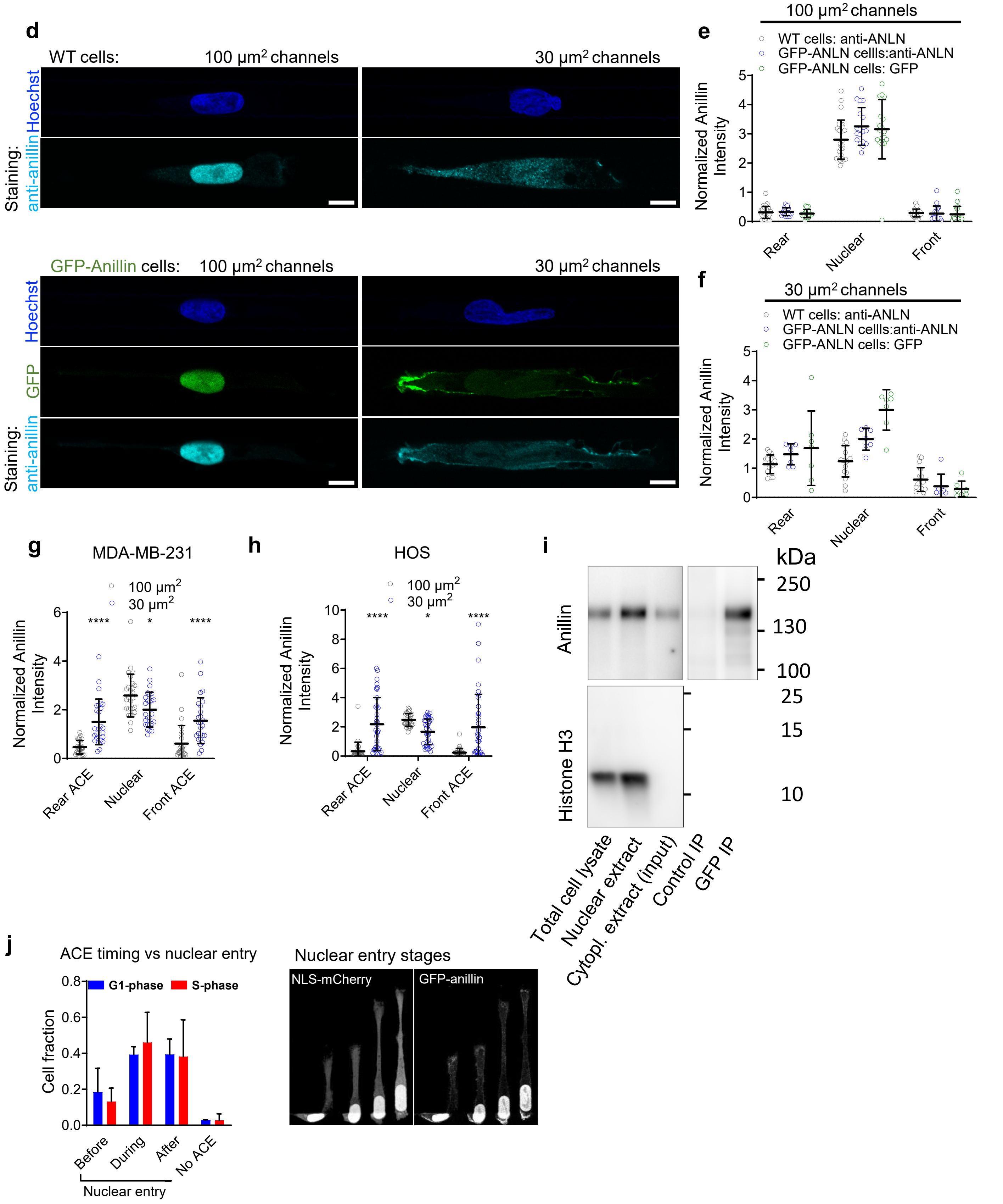

Supplemental Figure 2

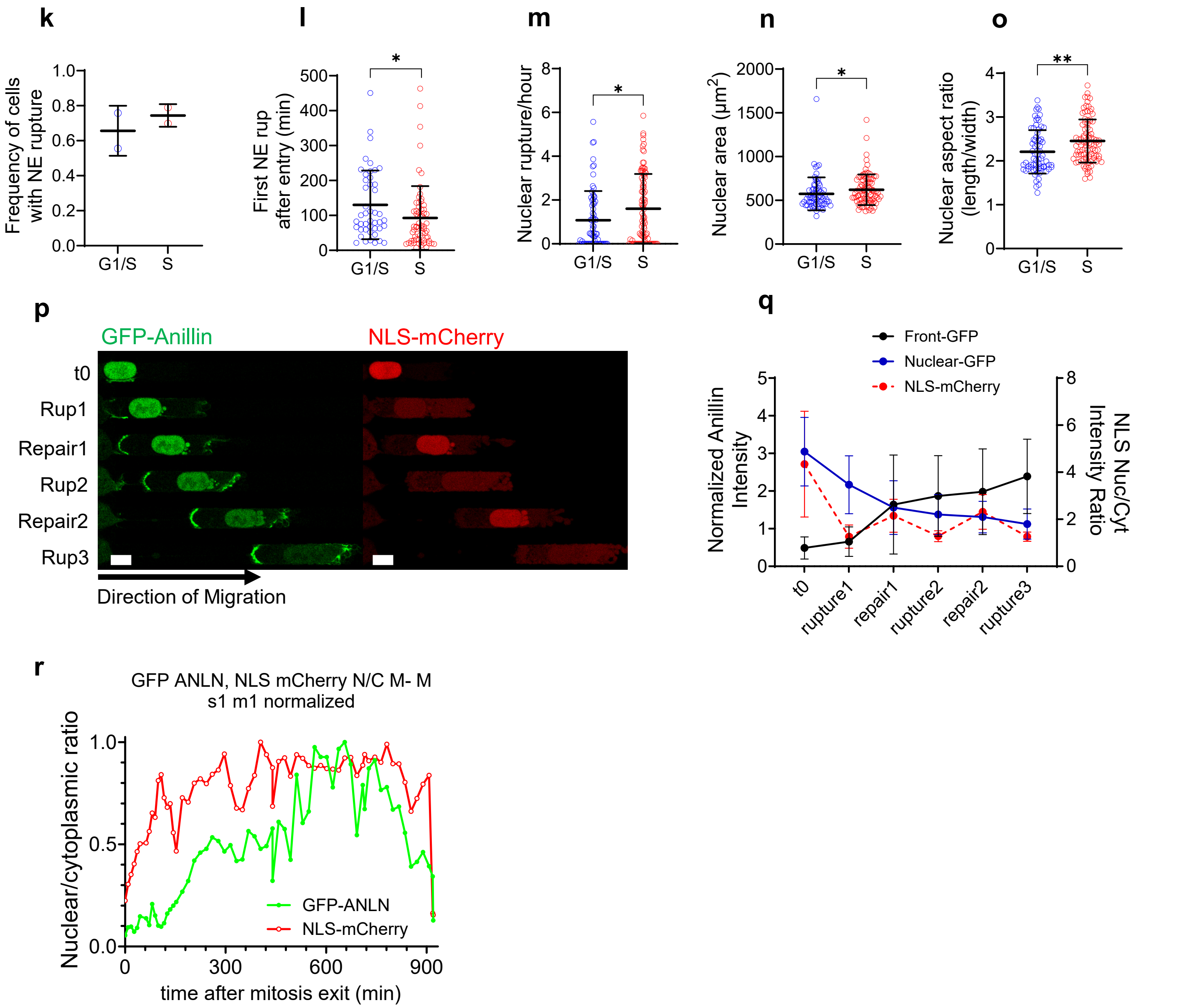

Supplemental Figure 2. Cytoplasmic anillin is detected in different cell types and becomes enriched upon NE rupture in confinement. (a,b) Cytoplasmic anillin exists in numerous cell lines: HT-1080, MDA-MB-231, HOS, A431, BRC-196 and HFF-1, with ACEs indicated by yellow arrows, as assessed by immunofluorescence staining (a) and quantification of cytoplasmic-to-nuclear intensity of anillin (b). Scale bar: 10 μm. **(c)** Schematic depicting the cell front, rear, and nuclear cell regions as used for quantification of anillin. **(d)** Representative images of HT-1080 WT or HT-1080 GFP-anillin (WT) cells migrating in moderately-confining or confining channels showing that GFP-anillin localization correlates with that of total anillin, as assessed by GFP-anillin (green) imaging and confirmed by immunofluorescence staining with Hoechst 33342 (blue) and anti-anillin antibody (cyan). Scale bars: 10 μm. **(e,f)** Quantification of localization of GFP-anillin and total anillin in HT-1080 WT and HT-1080 GFP-anillin (WT) cells migrating in 100 μm^2 (e) or 30 μm^2 channels (f). **(g,h)** Quantification of GFP-anillin localization in MDA-MB-231 (g) or HOS (h) cells migrating in 100 μm^2 and 30 μm^2 channels. **(i)** Representative co-immunoprecipitation showing RhoA and anillin interaction in the cytoplasm, as indicated by presence of anillin in GFP-pulldown of lysates from HT-1080 cells expressing the constitutively active RhoA construct GFP-RhoA(Q63L). **(j)** Quantification of frequency of cells with no ACEs or ACEs forming before, during, or after nuclear entry into confining channels. Representative images of nuclear entry stages on the right (n≥69 cells from 2 experiments). **(k,l)** Comparison of nuclear rupture frequency (k) and timing of first NE rupture following cell entry into confinement (l) between G1/S and S-phase synchronized cells. (n≥69 cells from 2 experiments). **(m-o)**

Hourly rate of NE rupture (m), nuclear area (n), and nuclear aspect ratio (o) of synchronized cells (n≥69 cells from 2 experiments). **(p)** Representative images of an unsynchronized cell experiencing repeated NE ruptures which resulted in intensifying ACEs. **(q)** Quantification of GFP-anillin intensity at the cell front and in the nucleus as well as NLS-mCherry nuclear-to-cytoplasmic intensity of unsynchronized cells in confinement (n=16 cells from 2 experiments). **(r)** Fluctuations of nuclear/cytoplasmic intensity ratios of GFP-anillin and NLS-mCherry after mitotic exit, showing anillin retention in the cytoplasm even after NLS-mCherry nuclear level has recovered. Values represent mean±SD. *p<0.05, ****p<0.0001 relative to 100 μm^2 as assessed by one-way ANOVA followed by Tukey's multiple comparisons test (c), two-way ANOVA followed by Sidak's test (h,i) or unpaired t-test (o) after log transformation (l,n) or Mann-Whitney test (m).

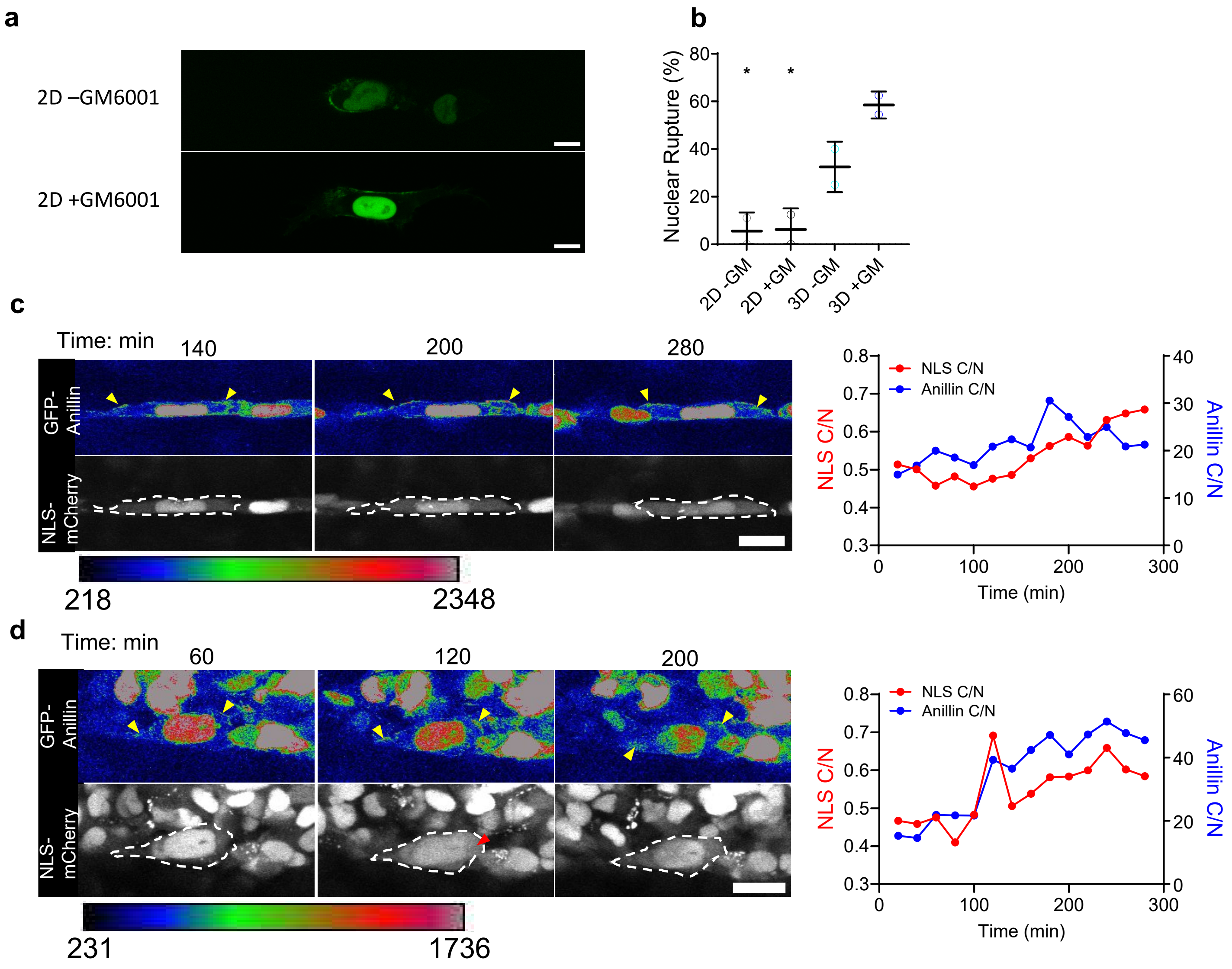

Supplemental Figure 3. Cytoplasmic anillin is detected in 2D and 3D hydrogels *in vitro* and in invading tumor cells *in vivo*. **(a)** Representative images of HT-1080 cells expressing GFP-anillin with ACEs on 2D collagen gels with or without GM6001. **(b)** Percentage of HT-1080 cells experiencing NE rupture events in 2D or 3D collagen gels with or without GM6001, as assessed by NLS-mCherry localization (n≥5 cells per experiment from 2 experiments). **(c)** Persisting anillin localization at the membrane (yellow arrow heads) monitored by time-lapse multiphoton microscopy (left panels). Scale bar: 20 µm. Right graph, time-dependent elevation of cytoplasmic/nuclear (C/N) ratio of GFP-anillin and NLS-mCherry intensity. **(d)** Example cell with recurrent NLS-mCherry leakage to the cytoplasm associated with increasing and persisting anillin positivity at cell extensions. Left panels, time-lapse multiphoton microscopy. The initial GFP-anillin signal at the cell edge increases in consecutive frames (yellow arrowheads). Red arrowhead, cytosolic NLS-mCherry peak. Scale bar: 20 µm. Right graph, time-dependent ratiometric analysis of cytoplasmic and nuclear intensity (C/N) of GFP-anillin and NLS-mCherry mean intensity. The peak of cytosolic NLS-mCherry (image panel, 120 min time-point) coincided with the short-lived ratiometric peak in the primary image (red arrowhead), whereas the concurrent anillin elevation was gradual. Values represent the mean±SD. *p<0.05 relative to 3D+GM assessed by one-way ANOVA followed by Tukey's multiple comparisons test.

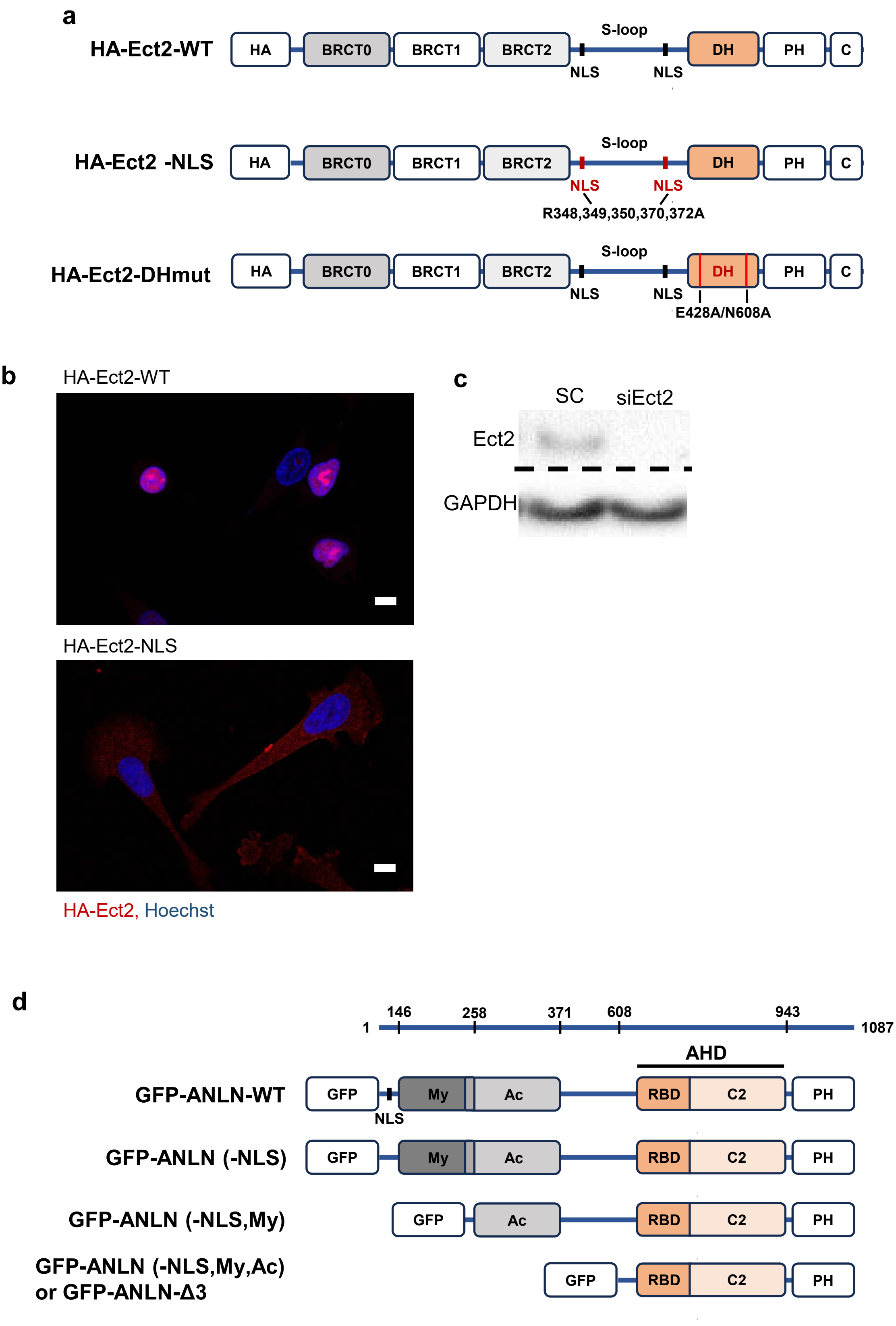

Supplemental Figure 4

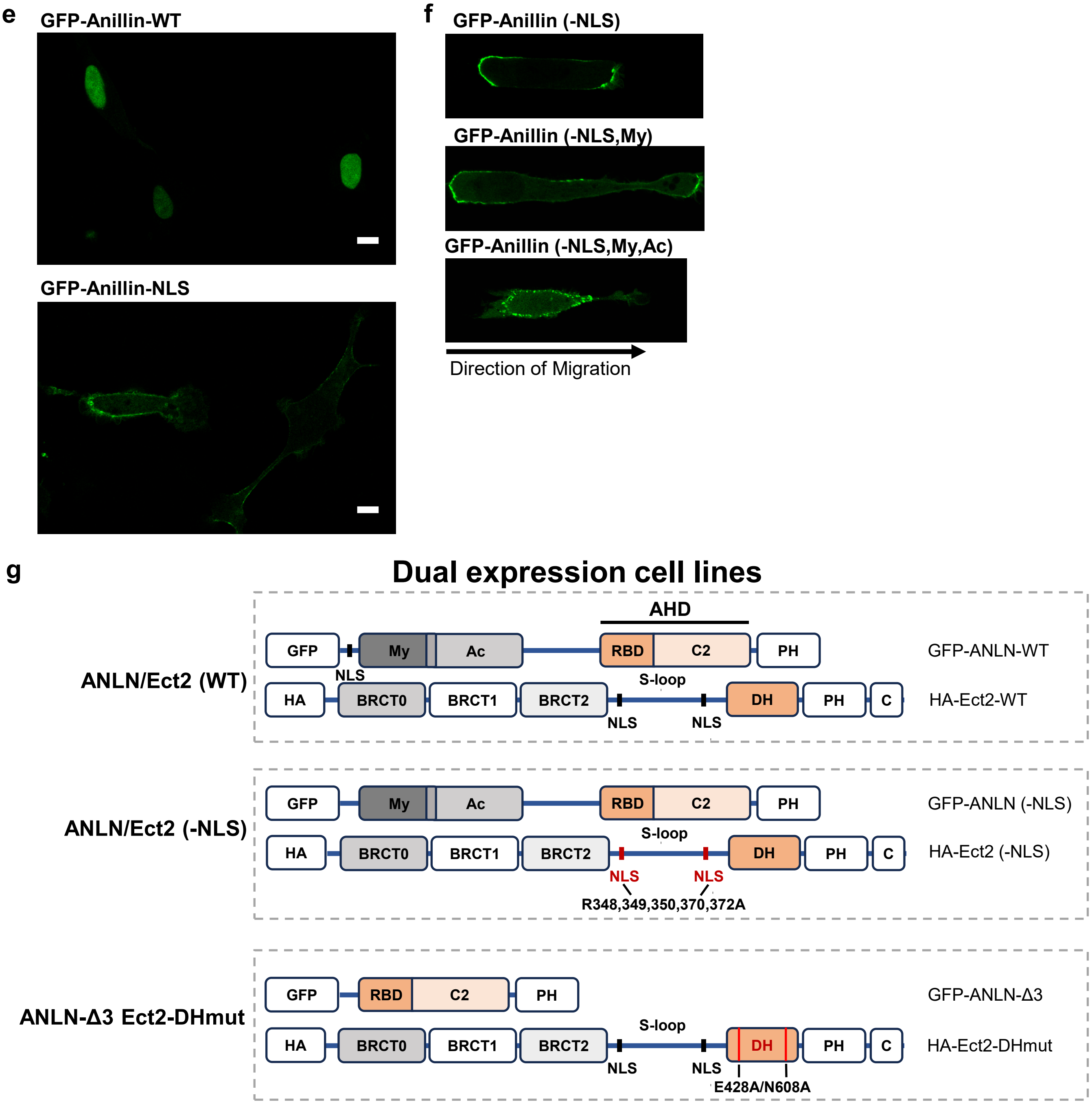

Supplemental Figure 4. Localization patterns of anillin and Ect2 wildtype and mutants. **(a)** Schematics depicting Ect2 mutant constructs (not in scale). Mutated regions are indicated in red. **(b)** Representative images of HT-1080 cells expressing HA-Ect2 (WT) or HA-Ect2 containing mutations in its NLS domain. **(c)** Western blot showing Ect2 knockdown efficiency via siRNA. GAPDH was used as a loading control. **(d)** Schematics depicting anillin mutant constructs with deletions of different binding domains used in this study (not in scale). **(e)** Representative images of HT-1080 cells expressing GFP-anillin (WT) or GFP-anillin with deletions of its NLS domain plated on 2D surfaces. **(f)** Representative images of HT-1080 cells expressing GFP-anillin with deletions of its NLS domain, or NLS and myosin binding domains, or NLS, myosin and actin binding domains, inside channels. **(g)** Schematics for the combination of anillin and Ect2 mutant constructs in the dual overexpression cell lines used in this study. Scale bars: 10 μm.

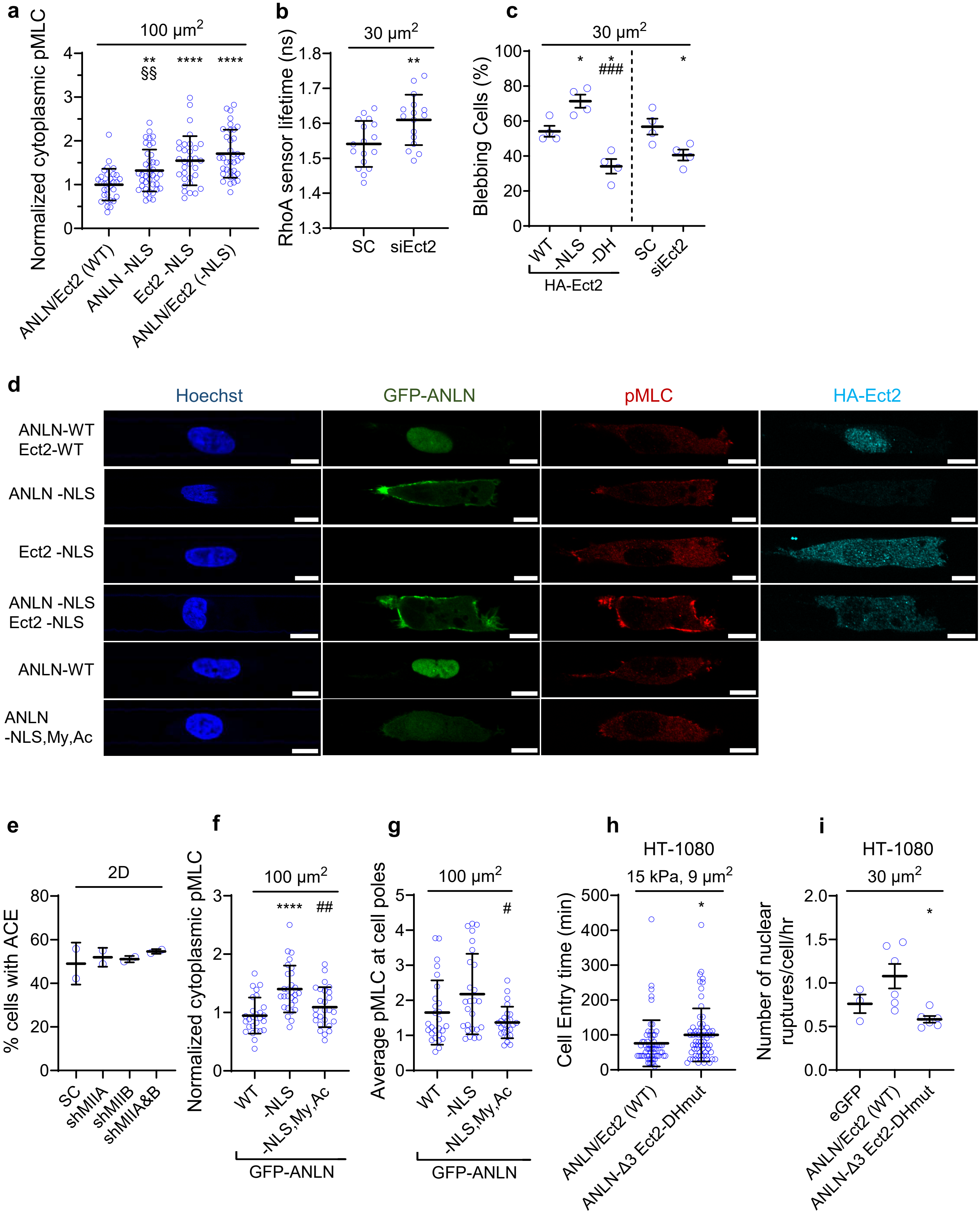

Supplemental Figure 5

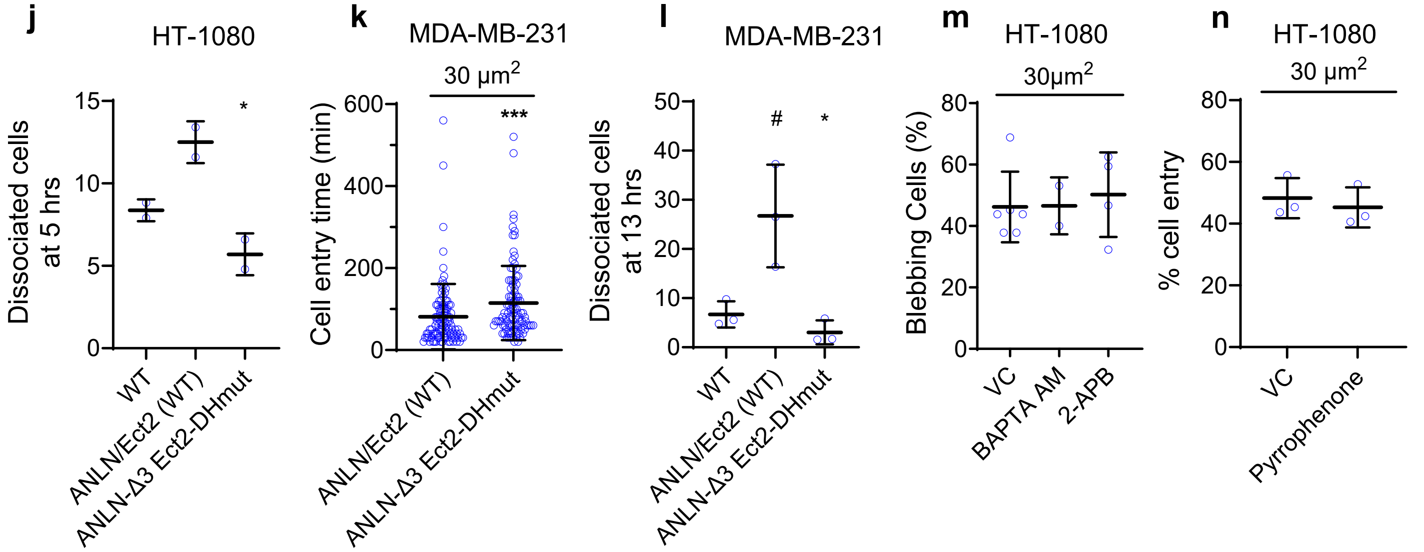

Supplemental Figure 5. Cytoplasmic enrichment of anillin and Ect2 mediated by NE rupture promotes RhoA/myosin II-dependent contractility and efficient cell entry into confinement. **(a)** Cytoplasmic pMLC intensity, as assessed from fixed and stained HT-1080 cells ectopically expressing either both GFP-anillin and HA-Ect2, or GFP-anillin or HA-Ect2 with a mutated NLS domain, or both GFP-anillin and HA-Ect2 with mutated NLS domains in moderately-confining channels (n≥32 cells from 3 experiments). **(b)** Donor fluorescence lifetime of RhoA activity biosensor RhoA2G for HT-1080 cells transiently transfected with either scramble control (SC) or siRNA against Ect2 (siEct2), as measured by FLIM (n≥16 cells from 2 experiments). **(c)** Percentage of blebbing cells in confining channels for HT-1080 cells ectopically expressing HA-Ect2 (WT), or HA-Ect2 with NLS mutations or HA-Ect2 with DH mutations. In separate experiments, the percentage of blebbing cells in confining channels was calculated for SC or siEct2 HT-1080 cells (n≥25 cells per experiment from 4 experiments). **(d)** Representative images of pMLC intensity for HT-1080 expressing the indicated constructs. **(e)** Percentage of scramble control (SC), MIIA-, MIIB-, or dual MIIA- and MIIB- knockdown HT-1080 cells displaying ACEs (n≥39 cells per experiment from 2 experiments). **(f,g)** pMLC cytoplasmic intensity (f) and its distribution at the poles (g), as assessed from fixed and stained HT-1080 cells ectopically expressing either GFP-anillin (WT) or GFP-anillin with a deleted NLS domain, or GFP-anillin with NLS, Myosin, Actin deletion (ANLN-Δ3) (n≥8 cells per experiment from 3 experiments). **(h)** Quantification of cell entry time for HT-1080 expressing GFP-anillin(WT)/HA-Ect2(WT) or the dual mutant during migration in compliant (15 kPa) tightly confining channels (n≥29 cells per experiment from 2 experiments). **(i)** Average number of nuclear ruptures per cell per hour for HT-1080 cells expressing eGFP (control), GFP-anillin (WT)/HA-Ect2 (WT) or the dual mutant during migration in PDMS-based confining channels (n≥33 cells per experiment from ≥3 experiments). **(j)** Number of cells dissociating from spheroids embedded in 3D collagen gels after 5 h for the following cells: HT-1080 WT, GFP-anillin (WT)/HA-Ect2 (WT) or the dual mutant cells (n≥ 5 spheroids per experiment from 2 experiments). **(k)** Quantification of cell entry time for MDA-MB-231 expressing GFP-anillin(WT)/HA-Ect2(WT) or GFP-anillin-Δ3 and HA-Ect2-DHmut (dual mutant) during migration in PDMS-based confining channels (n≥50 cells per experiment from 2 experiments). **(l)** Number of cells dissociating from spheroids embedded in 3D collagen gels after 13 h for the following MDA-MB-231 cells: WT, GFP-anillin (WT)/HA-Ect2 (WT) or the dual mutant cells (n≥12 spheroids per experiment from 3 experiments). **(m)** Percentage of blebbing cells in confining channels for HT-1080 cells treated with vehicle, BAPTA AM (25 μM) or 2-APB (100 μM) (n≥15 cells per experiment from ≥2 independent experiments). **(n)** Percentage of cells that entered confining channels for mCherry-tagged HT-1080 cells expressing GFP-anillin (WT) and HA-Ect2 (WT) under vehicle control (VC) or $cPLA_2$ inhibition with pyrrophenone (n≥183 cells per experiment from 3 experiment). The HT-1080 and MDA-MB-231 cell lines used in (j,l,m) express mCherry and were sorted for both GFP and mCherry positive cells. Values represent the mean±SD. *p<0.05, **p<0.01, ***p<0.001, ****p<0.0001 relative to GFP-anillin WT//HA-Ect2 WT (a, h-l), SC (b,c) or HA-Ect2 WT (c) or GFP-anillin WT (f). #p<0.05, ##p<0.01, ###p<0.001 relative to HA-Ect2 with mutated NLS (c), GFP-anillin lacking NLS (f,g), mCherry-tagged WT (l). Significance was assessed by one-way ANOVA followed by Tukey's multiple comparisons test (c, f,i,j,l) after log transformation (a,g), unpaired t-test (b, c (SC vs siEct2)) after log transformation (h,k).

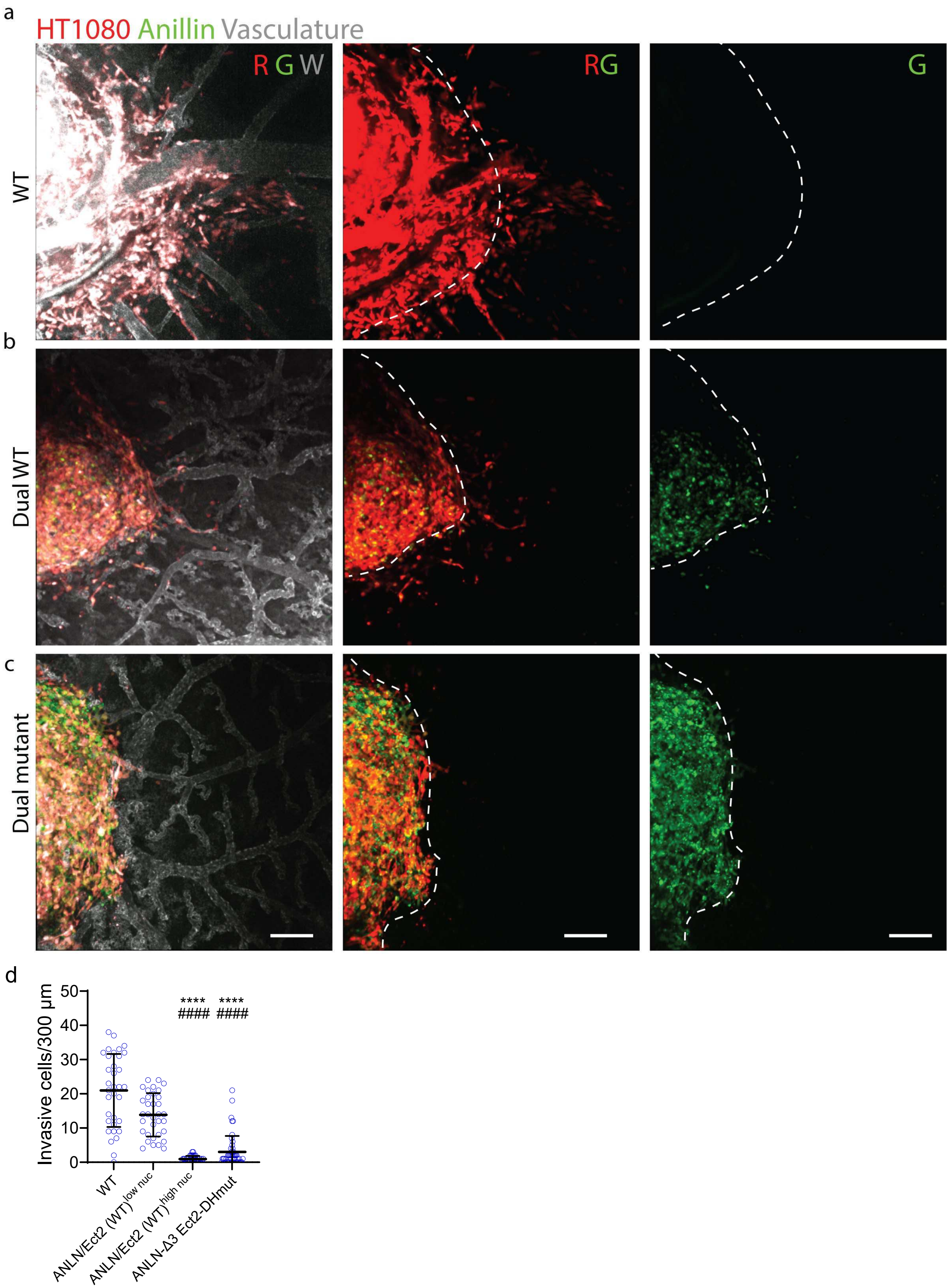

Supplemental Figure 6. Dual anillin and Ect2 mutation suppresses cell invasion in a chick embryo model. **(a-c)** Representative overview (10x) images showing primary tumors formed by mCherry-tagged

HT-1080 WT cells (a), mCherry-tagged HT-1080 cells expressing GFP-anillin (WT) and HA-Ect2 (WT) (b) or dual mutant (c) with maximum intensity projection views. Dashed white lines denote arbitrary tumor invasion fronts. Scale bar: 100 µm. **(d)** Quantification of the number of invasive cells at the primary tumor invasive zone for HT-1080 WT, GFP-anillin (WT)/HA-Ect2 (WT) (dual WT) with low nuclear anillin or high nuclear anillin, or dual mutant cells (n≥34 cells from ≥10 animals). Values represent the mean±SD. ****p<0.0001 relative to WT; ####p<0.0001 relative to dual $WT^{low\ nuc}$ assessed by Kruskal-Wallis followed by Dunn's multiple comparisons test.
